# Research progress in animal models of dry eye disease: Types, mechanisms, and application prospects

**DOI:** 10.1002/ame2.70192

**Published:** 2026-04-03

**Authors:** Jinshen Liu, Jiaqi Chen, Zhonghui Li, Jiamin Zhou, Hui Zhang, Yujia Yu, Feng Cheng, Jie Bao, Chunhan Feng, Xiangqu Yu, Zhao Xia, Rao Ding, Xiang Li

**Affiliations:** ^1^ Eye School of Chengdu University of Traditional Chinese Medicine Chengdu Sichuan Province China; ^2^ Ophthalmology Department Hospital of Chengdu University of Traditional Chinese Medicine Chengdu Sichuan Province China

**Keywords:** 3R principles, animal models, aqueous deficiency, dry eye disease, evaporative dry eye, inflammation, neuropathy, preclinical research

## Abstract

Dry eye disease (DED) is a prevalent and complex multifactorial ocular surface disorder, leading to significant visual discomfort and diminished quality of life. Animal models are indispensable tools for investigating DED pathology and evaluating therapeutic interventions. This review aims to systematically summarize the primary types of animal models of DED, detail their establishment methods and pathophysiological features, explore their value in elucidating key mechanisms, critically assess their strengths and limitations, and discuss their application prospects. A comprehensive literature search was conducted in electronic databases, including PubMed, Web of Science, and Google Scholar, with a primary focus on literature published within the past decade. Diverse animal models successfully replicate core features of different DED subtypes. Aqueous‐deficient models (e.g., surgical excision, scopolamine) mimic tear volume reduction and lacrimal gland inflammation. Evaporative models (e.g., desiccating stress, benzalkonium chloride) effectively simulate tear film lipid layer dysfunction and increased evaporation. Neurogenic models reveal the critical role of neural regulation and neuroinflammation, whereas multifactorial models (e.g., autoimmune, environment‐drug combinations) offer high clinical relevance by integrating multiple pathogenic factors. These models have been instrumental in identifying key inflammatory signaling pathways (e.g., NF‐κB), immune cell infiltration dynamics, and corneal nerve morphological and functional changes. Animal models are crucial for advancing our understanding of DED pathogenesis and developing novel therapies. The rational selection and application of appropriate models, based on research objectives, are paramount for enhancing translational relevance. These efforts are essential for bridging the translational gap between preclinical research and clinical application.

## Graphical abstract

Pivotal multifactorial animal models for translational dry eye disease research.

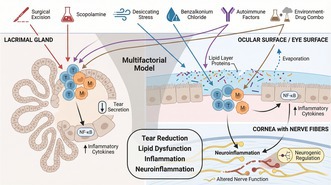

## 1 INTRODUCTION

Dry eye disease (DED) is a prevalent, multifactorial disorder of the ocular surface system, characterized by symptoms of discomfort, visual disturbance, tear film instability, and potential ocular surface damage.^1^ It represents a significant and growing global public health concern, adversely affecting quality of life and productivity, and imposing a substantial economic burden.^2^, ^3^ The core pathophysiological mechanism involves a self‐perpetuating vicious cycle encompassing tear film instability, hyperosmolarity, inflammatory responses, and neurosensory dysfunction.^4^, ^5^

A stable tear film, essential for ocular surface health, is maintained by the integrated function of the lacrimal glands (aqueous secretion), meibomian glands (lipid layer), conjunctival goblet cells (mucin), and the corneal/conjunctival epithelia, all under precise neural regulation.^6^ Disruption in any component can lead to DED, clinically categorized as aqueous‐deficient, evaporative, or a combination of both.^7^, ^8^ Understanding the intricate mechanisms within this “neuro‐immune‐epithelial” network—such as the role of inflammatory mediators like IL‐1β, IL‐6, and TNF‐α and the interplay with neurosensory pathways—is fundamental for developing targeted therapies.^9^, ^10^

In this pursuit, preclinical animal models are indispensable. They provide a crucial in vivo platform to dissect pathogenic cascades over time, validate molecular targets, and conduct initial efficacy and safety assessments of therapeutic interventions.^11^ A diverse array of models have been established through methods like lacrimal gland excision, pharmacological inhibition (e.g., scopolamine), chemical exposure (e.g., benzalkonium chloride [BAC]), and environmental desiccation stress.^12^, ^13^, ^14^ These models successfully replicate key features of human DED, including reduced tear secretion, shortened tear breakup time (TBUT), corneal epithelial damage, and immune cell infiltration.^15^, ^16^ For instance, animal studies have been instrumental in linking tear film instability to molecular changes such as altered occludin/MUC5AC co‐localization and in demonstrating the clinical predictive potential of artificial intelligence (AI)‐based diagnostics.^17^, ^18^

However, it is critical to recognize that no single animal model can fully recapitulate the complexity, heterogeneity, and chronicity of human DED. Physiological, anatomical, and immunological differences between species mean each model captures only selected facets of the disease.^12^, ^13^, ^14^ This inherent limitation necessitates a rational, question‐driven selection of models. Furthermore, ethical research mandates adherence to the 3Rs principles (Replacement, Reduction, Refinement). This drives innovation toward complementary advanced in vitro platforms—such as 3D human cell‐based ocular surface models and microfluidic systems—for early‐stage screening and mechanistic studies, refining hypotheses before animal testing.^19^, ^20^, ^21^

Within this conceptual and ethical framework, the present review aims to provide a comprehensive and integrative analysis of preclinical DED models. Moving beyond mere cataloging, we (1) propose a refined classification system (aqueous‐deficient, evaporative, neurogenic, multifactorial) aligned with clinical subtyping; (2) synthesize evidence across inflammation, neural injury, and immune dysregulation into a cohesive pathophysiological network; and (3) critically evaluate translational gaps to offer strategic directions for model optimization and future research, ultimately contributing to the advancement of precision medicine in DED management.

## 2 LITERATURE SEARCH STRATEGY

**FIGURE 1:**
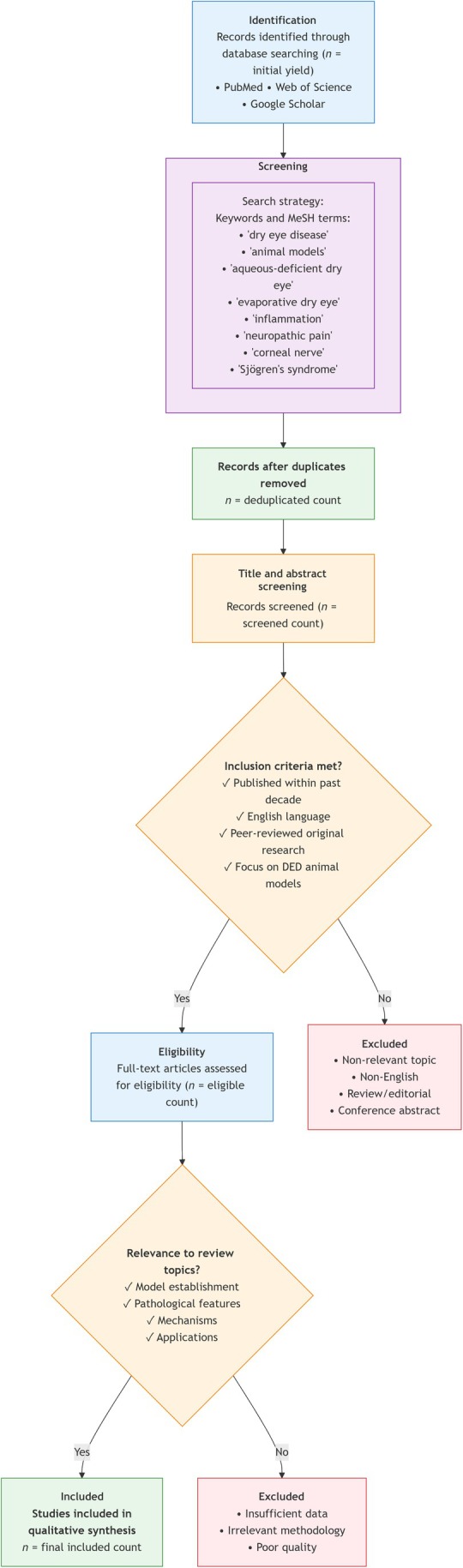
PRISMA‐style flowchart.

A systematic and comprehensive literature search was conducted to identify all relevant studies on animal models of DED. The search was performed across three major electronic databases: PubMed, Web of Science, and Google Scholar. The primary focus was on literature published within the last decade to ensure the review's contemporary relevance. The search strategy employed a combination of targeted keywords and Medical Subject Headings (MeSH) terms, including: “dry eye disease,” “animal models,” “aqueous‐deficient dry eye,” “evaporative dry eye,” “inflammation,” “neuropathic pain,” “corneal nerve,” and “Sjögren's syndrome.” Boolean operators (AND, OR) were used to combine these terms effectively. The selection process followed a structured, multistage screening protocol to ensure the inclusion of high‐quality, pertinent studies. As detailed in Figure 1, identified records were first deduplicated. The titles and abstracts of the remaining articles were then screened against predefined inclusion criteria: publication in English within the past 10 years, peer‐reviewed original research, and a primary focus on the establishment, characterization, or application of DED animal models. Articles meeting these criteria underwent full‐text assessment for their direct relevance to the core themes of this review: model classification, establishment methods, pathological mechanisms (e.g., inflammation, neural injury), and translational applications. Studies were excluded if they were review articles and conference abstracts, lacked primary data, or did not specifically address animal models of DED. This rigorous process culminated in the final set of studies included for qualitative synthesis and data extraction in this review.

## 3 PRECLINICAL ANIMAL MODELS OF DED

### 3.1 Classification and establishment methods

**FIGURE 2:**
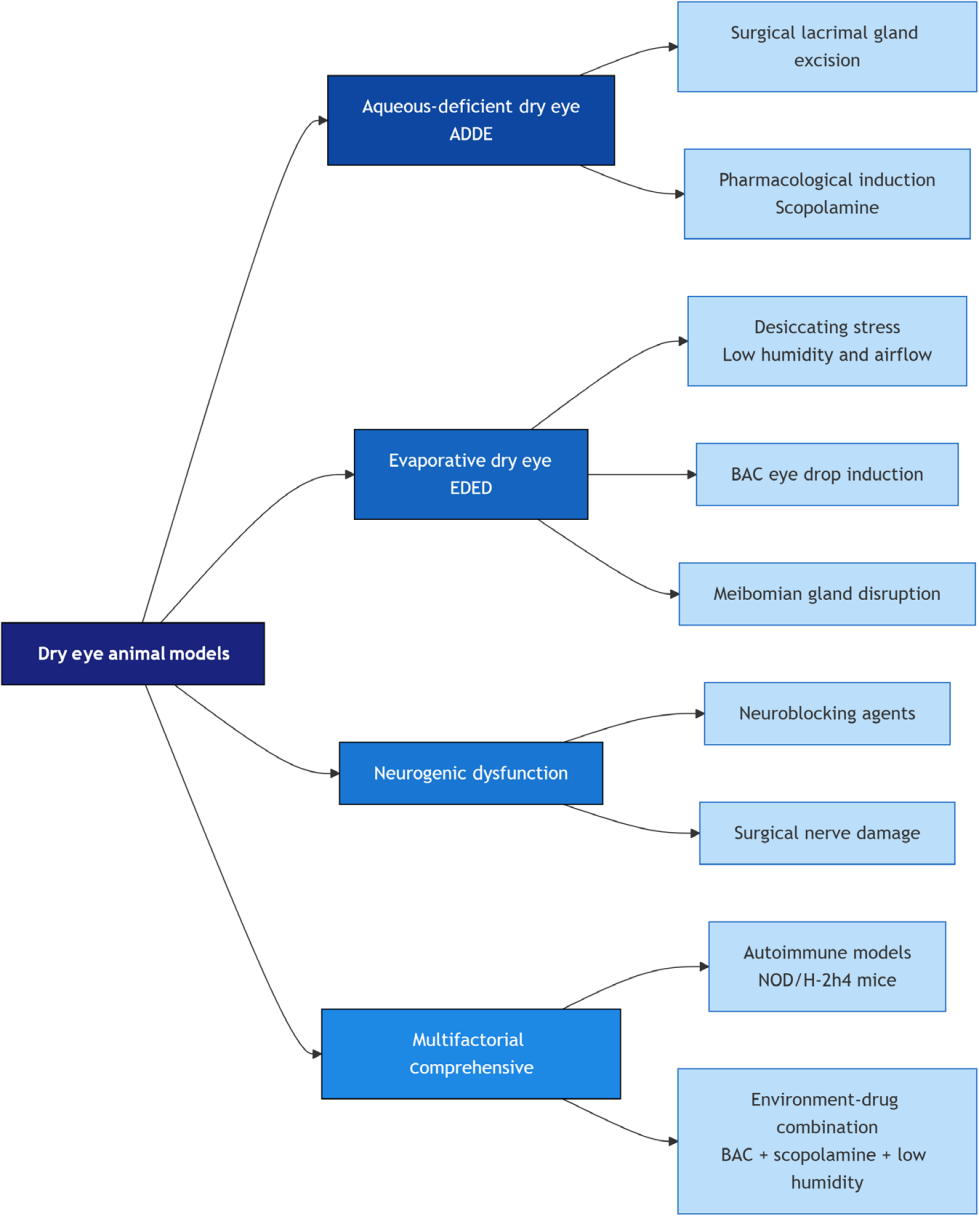
Types of dry eye animal models.

DED animal models can be systematically categorized into four main types based on the primary pathological mechanism they simulate: aqueous‐deficient, hyperevaporative, neurogenic dysfunction, and multifactorial comprehensive models. Figure 2 illustrates this categorization and the specific methodologies employed for model establishment.

#### 3.1.1 ADDE model

The aqueous‐deficient dry eye (ADDE) model primarily aims to replicate the pathological state of reduced tear volume in DED by impairing lacrimal gland secretion. Common techniques include the surgical removal of the lacrimal gland and pharmacological suppression of tear production.^22^ Surgical models often involve bilateral excision of the extraorbital lacrimal gland (ELG) and intraorbital lacrimal gland (ILG), an approach frequently used in mice. Pharmacological models typically employ sustained administration of scopolamine, a muscarinic antagonist that effectively inhibits tear secretion, commonly applied in rats.^23^

In the mouse bilateral lacrimal gland excision model, animals develop pronounced dry eye symptoms within 2 weeks post‐surgery, featuring significantly decreased tear volume, irregular corneal epithelium, positive fluorescein staining, and corneal neovascularization. Histological analyses reveal aberrant corneal epithelial differentiation, characterized by downregulated expression of the corneal epithelial markers K12 and Pax6, and upregulated Sprr1b expression. Concurrently, increased Ki67 staining indicates enhanced corneal epithelial cell proliferation, suggesting a state of concurrent damage and repair. These epithelial pathologies are accompanied by inflammatory cell infiltration, reflecting localized inflammation secondary to tear insufficiency.^23^ This model is a valuable tool for investigating corneal and conjunctival pathologies arising from impaired tear secretion.

The rat scopolamine‐induced model pharmacologically suppresses lacrimal gland function, leading to reduced tear secretion and subsequent corneal and conjunctival inflammation. It enables the comprehensive assessment of lacrimal gland function and ocular surface pathology, presenting clinical features of lacrimal gland dysfunction and providing a robust platform for exploring ADDE mechanisms and therapies.^24^

Key pathological hallmarks of ADDE models include reduced tear volume and diminished tear film stability, culminating in corneal epithelial damage and inflammation. Research indicates that aqueous deficiency not only compromises the physical stability of the tear film but also initiates a cascade of local immune activation, fostering chronic inflammation that exacerbates corneal injury. For instance, lacrimal gland excision in mice alters corneal nerve morphology and function, upregulating inflammation‐related markers like CGRP and TRPV1 in neuronal cells, underscoring the role of neuroinflammation in ADDE pathogenesis.^25^ Furthermore, lacrimal gland dysfunction often involves neural regulatory imbalances. Studies have identified that the sympathetic nervous system modulates tear secretion via the NA‐Adra1a‐Ucp2 pathway, and its blockade ameliorates dry eye symptoms, pointing to neural regulation as a significant research avenue in ADDE models.^26^

ADDE models are particularly suited for studying the pathogenesis of tear secretory failure and associated inflammation, especially in autoimmune contexts like Sjögren's syndrome (SS). SS patients frequently experience severe aqueous deficiency due to lacrimal gland involvement. Animal models simulating this state via lacrimal gland excision effectively recapitulate clinical manifestations. Lacrimal gland excision‐based models have also unveiled substantial alterations in gene expression and the proteome, reflecting the profound impact of chronic inflammation on the lacrimal gland.^27^ Moreover, the elevation of inflammatory cytokines such as IL‐20 in DED patients and their functional role in models inform novel strategies for targeted anti‐inflammatory therapy.^28^

Currently, ADDE models find broad application in DED research, including drug screening, mechanistic dissection, and novel therapy evaluation. For example, preclinical studies and early‐phase clinical trials involving mesenchymal stem cell (MSC) injections into the lacrimal gland demonstrate safety and potential for improving tear secretion, underscoring the translational relevance of ADDE model‐based research.^29^ Furthermore, emerging therapeutic approaches like salivary gland transplantation, neural modulation, and bioengineered lacrimal gland regeneration also rely on valid ADDE animal models for verification.^30^

In conclusion, aqueous‐deficient dry eye animal models, established via lacrimal gland excision or pharmacological tear suppression, successfully mimic core ADDE pathological features, including reduced tear volume, corneal epithelial damage, and local inflammation. These models provide a solid foundation for elucidating DED pathogenesis and assessing novel therapeutic strategies, remaining indispensable in the DED research arsenal.^23^, ^24^, ^30^

#### 3.1.2 EDED model

Evaporative dry eye (EDED) is a major DED subtype whose core pathology involves dysfunction of the tear film lipid layer, leading to excessive tear evaporation, subsequent corneal surface inflammation, and tear film destabilization.^31^ Animal models of EDED are typically induced by environmental desiccation, eyelid dysfunction, or chemical stimulation to mimic the clinical EDED phenotype.^32^ Representative models include the desiccating stress model, the BAC induction model, and the lacrimal gland excision combined with environmental stress model. Comparative studies between scopolamine and BAC‐induced evaporative models revealed that scopolamine primarily induces lacrimal gland and corneal inflammation, with dry eye symptoms persisting post‐drug discontinuation, highlighting its utility for evaluating long‐term treatment efficacy.^33^, ^34^

The desiccating stress model induces increased tear film evaporation by housing animals in controlled low‐humidity and/or windy environments, resulting in corneal and conjunctival inflammation. Studies confirm that low‐humidity exposure reliably triggers tear film instability and corneal epithelial damage, replicating hallmark EDED clinical manifestations.^33^ The BAC induction model exploits the ocular surface toxicity of BAC to disrupt the integrity of the tear film lipid layer, augmenting tear evaporation and inducing corneal inflammation. Its advantages include procedural simplicity and reproducibility, making it suitable for assessing anti‐inflammatory and reparative drugs.^33^ The lacrimal gland excision combined with environmental stress model incorporates elements of both aqueous deficiency and increased evaporation, simulating mixed dry eye where the evaporative component is predominant, thereby facilitating the study of tear film lipid layer defects and tear dynamics.^33^

Pathologically, EDED models are characterized by significantly increased tear evaporation rates, active corneal and conjunctival inflammation, and shortened TBUT. The integrity and function of the lipid layer are compromised, directly contributing to tear film instability, a defining feature of EDED.^35^, ^36^ Model animals often exhibit reduced tear film lipid layer thickness, impaired meibomian gland secretion, corneal epithelial cell damage, and inflammatory cell infiltration.^36^, ^37^

These models are primarily utilized to investigate the stability of the tear film lipid layer and the molecular basis of its dysfunction, particularly the relationship between MGD and tear film abnormalities.^38^, ^39^ For instance, the BAC‐induced model is routinely used to evaluate the efficacy of novel lipid‐based eye drops or anti‐inflammatory therapies in improving tear film stability, serving as a key tool for researching inflammation‐mediated EDED treatments.^40^, ^41^ Additionally, environmental dry eye models support the assessment of interventions targeting lifestyle and environmental factors, providing experimental evidence for clinical prevention and management strategies.^42^, ^43^

In summary, evaporative dry eye animal models, established through environmental desiccation, chemical stimulation, or lacrimal gland interference, successfully replicate the core pathological features of clinical EDED. Their primary manifestations include increased tear evaporation, corneal inflammation, and shortened TBUT, rendering them appropriate for studying tear film lipid layer stability and MGD mechanisms. They constitute essential experimental platforms for pathophysiological investigation and therapeutic development for EDED.^36^, ^38^, ^39^

#### 3.1.3 Neurogenic dysfunction model

Neurogenic dysfunction models seek to elucidate the role of neural mechanisms in DED pathophysiology by simulating impaired neural regulation, primarily through nerve blockade or injury.^44^ Representative approaches include the use of neuroblocking agents and surgical damage to lacrimal gland innervation. These models replicate reduced tear secretion, sensory dysfunction, and associated neuroinflammation resulting from compromised innervation, mirroring the neurogenic dysfunction observed in DED patients.^45^ Nerve injury‐induced tear deficiency is largely attributed to an imbalance in the neural control of the lacrimal gland, particularly involving parasympathetic and sympathetic input. A study employing extraorbital lacrimal gland excision in mice, modeling ADDE, demonstrated impaired lacrimal gland function and nerve regeneration, accompanied by increased neuroinflammation and immune cell infiltration, indicating that neural damage exacerbates local inflammation beyond affecting tear secretion alone.^16^ Furthermore, corneal sensory nerves are compromised post‐injury, manifesting as significant reductions in nerve fiber length (NFL) and nerve fiber density (NFD), leading to sensory abnormalities and hyperalgesia. In vivo confocal microscopy (IVCM) reveals structural alterations in corneal nerves of DED patients, including decreased nerve length and branch points, alongside increased tortuosity, reflecting the extent of neural damage.^46^

Mechanistically, neuroinflammation following nerve injury is a key contributor to sensory dysfunction. Injury‐induced neuroinflammation can activate the NF‐κB/IL‐6 signaling pathway, perpetuating an inflammatory state that delays corneal epithelial repair and nerve regeneration, thereby worsening DED pathology.^16^ Neuroinflammation also promotes peripheral nociceptive sensitization, contributing to persistent ocular discomfort and pain. Neurogenic injury models have also highlighted the role of neuropeptides; for instance, vasoactive intestinal peptide (VIP) levels increase significantly after laser refractive surgery, suggesting its function as a neuroprotective and anti‐inflammatory molecule in mitigating post‐neural injury dry eye symptoms.^45^

Neurogenic dysfunction models hold significant utility in DED research. They facilitate a deeper understanding of how neural regulatory abnormalities contribute to DED onset, revealing the complex interplay within the neuro‐immune‐epithelial network and aiding systematic pathological analysis. By simulating nerve injury and its sequelae, these models provide a platform for identifying therapeutic targets focused on neuroinflammation and nerve regeneration. For example, topical nerve growth factor (NGF) application has been shown to promote corneal nerve regeneration and improve DED symptoms, demonstrating therapeutic promise.^47^ Similarly, topical collagen mimetic peptide (CMP) can effectively repair damaged corneal nerve networks, mitigate neurodegenerative changes, and facilitate functional recovery.^48^ The development of such strategies is contingent upon the availability and refinement of neurogenic dysfunction models.

In conclusion, neurogenic dysfunction models, through neural blockade or injury, successfully recapitulate neuroinflammation, sensory abnormalities, and hyperalgesia in DED. They not only advance our understanding of DED's neural underpinnings but also provide critical experimental evidence and theoretical support for discovering pain‐related therapeutic targets and developing neuroprotective and regenerative treatments, establishing themselves as key instruments in basic and translational DED research.^49^, ^50^

#### 3.1.4 Multifactorial comprehensive models

Multifactorial comprehensive models represent a more sophisticated and clinically relevant category of DED animal models. They integrate multiple pathogenic factors—such as aqueous deficiency, increased tear evaporation, and immune dysregulation—to more holistically simulate the complex pathological landscape of DED.^51^ These models capture not only the pathologies induced by individual factors but also reflect the intertwined, multi‐mechanistic progression characteristic of the disease, thus holding considerable value for investigating DED pathogenesis and evaluating comprehensive treatment strategies.^52^

Prominent multifactorial models include autoimmune‐related models and environment‐drug combination models. Autoimmune models, particularly the SS mouse models like the non‐obese diabetic (NOD) mouse and its genetically modified strains (e.g., NOD.H‐2h4, Aire−/−), are widely used to simulate SS‐associated DED. These models exhibit tear secretory dysfunction, immune cell infiltration, and significant inflammation, often with marked gender disparities (earlier onset and greater severity in females), effectively mirroring the clinical gender bias and pathology of SS.^53^ For instance, NOD.H‐2h4 double‐knockout mice display significant tear reduction, corneal damage, and lacrimal gland inflammation, with infiltrating immune cells predominantly comprising B cells, alongside helper T cells and macrophages.^53^

Environment‐drug combination models induce aqueous deficiency and ocular surface inflammation by concurrently applying desiccating environmental stressors (e.g., low humidity, airflow) and pharmacological agents (e.g., BAC, scopolamine), simulating DED pathology driven by the combined influence of diverse environmental and drug factors encountered clinically.^12^, ^54^ The pathological presentations of these models are complex, encompassing reduced tear volume, tear film instability, corneal epithelial damage, and immune‐mediated inflammation. Drug‐induced components, such as the BAC model, demonstrate corneal epithelial injury, elevated tear osmolarity, NLRP3 inflammasome activation, and transforming growth factor (TGF‐β) signaling anomalies, reflecting inflammation‐driven pathological processes.^55^

The pathological spectrum of multifactorial models spans tear dynamics disruption, ocular surface structural impairment, immune inflammation activation, and neurogenic dysfunction, enabling a relatively comprehensive recapitulation of DED pathogenesis. In autoimmune models, aberrant expression of cytokines like IL‐6 and IL‐17, along with T‐cell subset imbalances, participates in disease progression.^56^, ^57^ Similarly, environment‐drug models consistently demonstrate interactions among oxidative stress, apoptosis, and immune responses.^58^, ^59^

Owing to their complex pathology and high clinical similarity, multifactorial models are ideal for researching integrated treatment strategies for DED. They are suitable not only for deciphering the multifaceted pathogenesis of DED but also for evaluating how novel drugs or therapies impact different pathological components. Examples include using autoimmune models to assess the efficacy of immunomodulators in suppressing lacrimal gland inflammation or employing environment‐drug models to test the therapeutic potential of antioxidants and anti‐inflammatory agents.^60^, ^61^ Additionally, non‐human primate (NHP) models induced via environmental stress provide a valuable platform for translational clinical research.^62^

#### 3.1.5 Evaluation of model consistency indicators

The consistency and reliability of DED animal models are typically assessed through standardized clinical and molecular indicators.^11^ Key evaluation metrics include corneal fluorescein staining (to quantify epithelial damage), TBUT (reflecting tear film stability), tear volume measurement (e.g., Schirmer's test or phenol red thread test), and tear quality analysis (e.g., osmolarity, inflammatory cytokine levels).^63^ These indicators are essential for validating model establishment, monitoring disease progression, and evaluating therapeutic interventions. Their systematic application across different model types enhances comparability and translational relevance.

Critically, the selection of these metrics is strongly supported by their established utility and correlation with disease state in human clinical research. For instance, large‐scale prospective cohort studies have mechanistically linked alterations in these very parameters to specific environmental etiologies. A multicenter study in China demonstrated that exposure to air pollutants like PM~2.5~ and O~3~ was significantly correlated with worsened Ocular Surface Disease Index (OSDI) symptom scores, shortened TBUT, increased corneal fluorescein staining (CFS), and elevated levels of tear inflammatory cytokines (e.g., IL‐6, IL‐8). This confirms that the key signs measured in animal models directly mirror the pathophysiological changes observed in human DED triggered by environmental stress.^64^ Furthermore, the centrality of these indicators is reflected in global clinical practice and trial design. An international survey of DED experts revealed that corneal fluorescein staining is the most frequently used method (92%) for assessing ocular surface damage, and evaluation of tear film stability (e.g., TBUT) and secretion (e.g., Schirmer's test) remains a cornerstone of clinical diagnosis.^65^ Similarly, a comprehensive analysis of DED clinical trials (2000–2024) identified corneal staining, TBUT, the OSDI, and Schirmer's test as the most commonly employed primary or secondary endpoints. This convergence between model assessment and clinical trial endpoints underscores their shared biological relevance and facilitates translational extrapolation.^66^

Therefore, employing this multi‐parametric panel in animal studies does not merely serve to quantify a model's phenotype; it anchors the experimental findings within a well‐defined clinical context. This practice enhances the comparability of results across different laboratories and, most importantly, builds a crucial bridge for translating mechanistic insights and therapeutic candidates from bench to bedside.

#### 3.1.6 Comparative practical considerations for model selection

When selecting an appropriate DED animal model, researchers must consider practical parameters beyond pathological mimicry. Key factors include modeling efficiency (time to stable phenotype), success/reproducibility rate, operational complexity, cost, and ethical considerations. For instance, although scopolamine‐induced ADDE in rats achieves consistent tear reduction within 5–7 days with high reproducibility, its phenotype may be less stable long term compared to surgical models.^24^ BAC‐induced EDED models offer rapid induction but can exhibit variable severity.^67^ Autoimmune models (e.g., NOD mice) provide high clinical relevance for SS‐DED but require longer development times and have inherent genetic variability.^68^ A decision matrix balancing research objectives (e.g., mechanistic study vs. drug screening), timeline, resources, and the specific DED subtype under investigation is therefore crucial for optimal model selection.

In summary, multifactorial comprehensive models, by incorporating multiple pathogenic factors like aqueous deficiency, increased evaporation, and immune abnormalities, offer a realistic simulation of the complex clinical DED phenotype. They are poised to play an increasingly vital role in future research aimed at deepening our understanding of DED pathogenesis and developing multitargeted therapeutic regimens, thereby providing a solid experimental and theoretical foundation for precision medicine in DED.

Table 1 provides a detailed comparison of the establishment methods, pathological correlates, practical parameters, and applicability of various dry eye animal models, serving as a practical reference for researchers to select appropriate models based on experimental objectives.

**TABLE 1:** Comparative analysis of preclinical dry eye animal models.

Model type	Common modeling methods	Simulated human pathology	Representative species	Major advantages	Major limitations	Modeling success rate	Phenotype stability period	Typical modeling duration
Aqueous‐deficient dry eye	Lacrimal gland surgery, scopolamine injection	Sjögren's syndrome, lacrimal gland dysfunction	Mouse, rat	Clear tear reduction; suitable for lacrimal gland inflammation studies	May not fully mimic progressive human lacrimal gland damage	>90% for scopolamine; variable for surgery	1–4 weeks	1–2 weeks
Evaporative dry eye	BAC eye drops, low humidity, meibomian gland disruption	Meibomian gland dysfunction, evaporative dry eye	Mouse, rabbit	Simple procedure; stable simulation of lipid layer defect	Potential non‐specific damage from drug toxicity	~80%–90% for BAC	1–3 weeks	3–14 days
Neurogenic dysfunction	Neuroblocking agents, trigeminal/lacrimal nerve surgery	Neurogenic dry eye, post‐surgical dry eye, corneal hypoesthesia	Mouse, rat	Reveals neuro‐immune‐epithelial interactions	High surgical skill required; model stability can vary	Variable, depends on surgical precision	1–4 weeks	1–3 weeks
Multifactorial comprehensive	NOD/H‐2 h4 mice (autoimmune), BAC + scopolamine+low humidity	Sjögren's syndrome, complex clinical dry eye	Mouse, non‐human primate	High clinical relevance; complex pathology	Modeling complexity; high cost; difficult variable control	High for genetic models; variable for combos	Weeks to months	Weeks to months

### 3.2 Major animal species: Advantages and limitations

**FIGURE 3:**
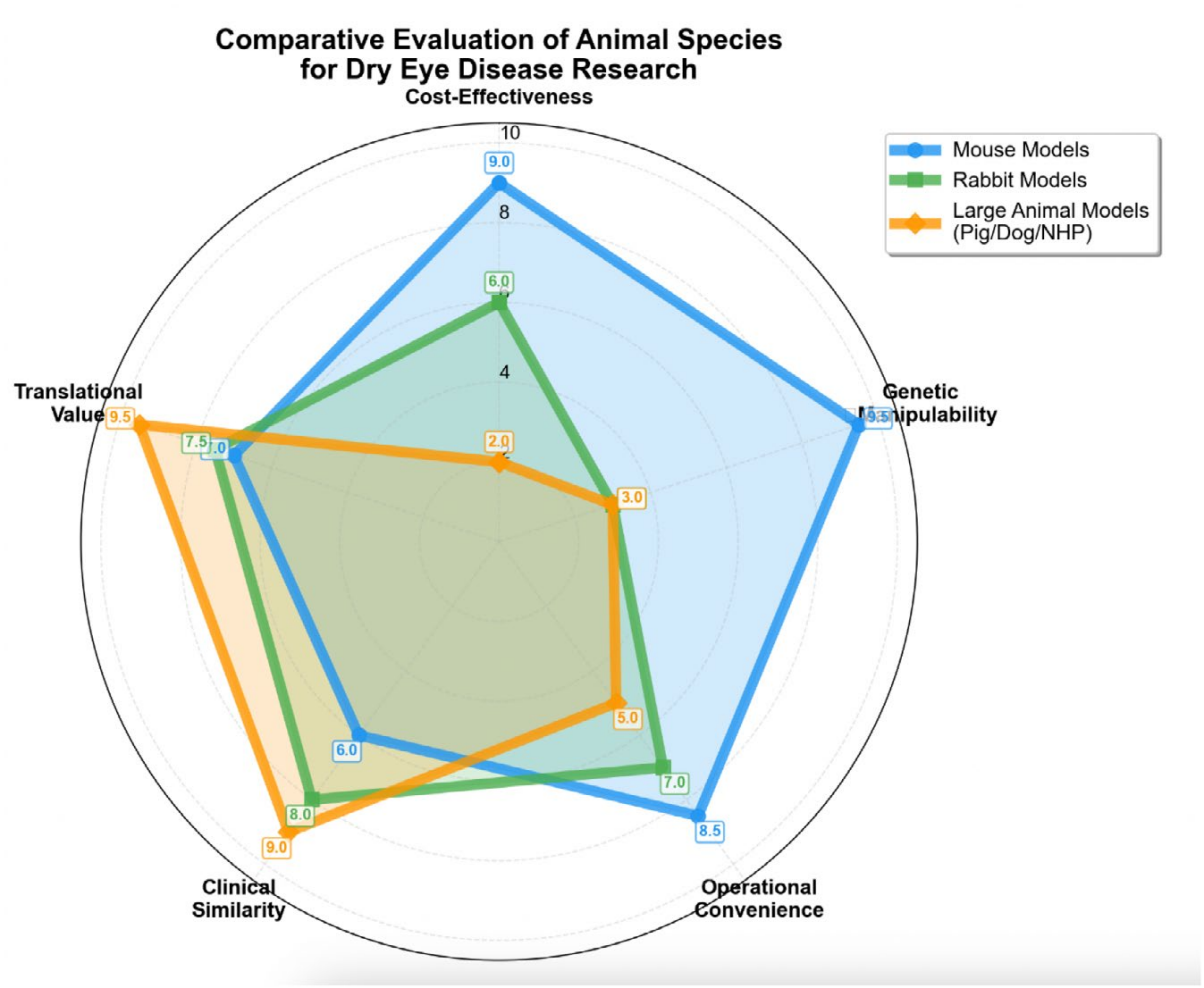
Radar chart comparing key characteristics of mouse, rabbit, and large animal models for dry eye research.

Figure 3 visually compares the characteristics of mouse, rabbit, and large animal models across five key dimensions—genetic manipulability, clinical similarity, cost‐effectiveness, operational convenience, and translational value—using a radar chart, thereby offering a scientific basis for model selection aligned with research goals. The scores (1–10) are semi‐quantitative assessments based on comparative analysis of the literature, where 1 represents the lowest suitability and 10 the highest. As visualized in Figure 3, mouse models excel in genetic manipulability (9.5/10) but score lower in clinical similarity (6/10), whereas large animals exhibit the opposite pattern.

#### 3.2.1 Mouse models

Mouse models are extensively utilized in DED research owing to their well‐defined genetic background, ease of genetic manipulation, and low cost. These models enable researchers to replicate human DED pathological features through techniques like gene knockout and transgenesis, facilitating deep exploration of disease mechanisms and potential therapies.^69^ For instance, the NOD mouse, along with Aire−/− and Id3 knockout strains, is commonly employed a as model for SS‐related dry eye, reflecting autoimmune lacrimal gland inflammation.^53^, ^69^ Moreover, genetically engineered mouse models for evaporative dry eye, such as Eda−/−, Sod1−/−, and Elovl1−/−, simulate MGD, further diversifying the DED animal model repertoire.^70^, ^71^

Despite their pronounced advantages for mechanistic studies and gene function validation, mouse models have inherent limitations. Their small eyeball size and limited tear volume result in some clinical manifestations that diverge from human DED. Techniques for measuring tear secretion and assessing ocular surface damage are more challenging in mice, and the intricate complexity of ocular surface neural and immune responses is difficult to fully replicate.^48^, ^72^ Furthermore, structural differences between murine and human lacrimal and meibomian glands mean that therapeutic efficacy observed in mice may not always translate directly to the clinic.^24^

In application, mouse models are primarily used to elucidate DED pathogenesis, immune cell involvement, and inflammatory signaling pathway regulation. For example, they have been instrumental in uncovering the heterogeneity of IL‐17‐producing Th17 cells and their associated signaling pathways in DED, informing targeted therapy development.^73^, ^74^ Research based on mouse models has also propelled the exploration of diverse treatments, including stem cell‐derived exosomes, anti‐inflammatory factors, gene therapy, and novel drug evaluation.^75^, ^76^, ^77^ Additionally, mouse models are used to assess physical therapies like multi‐wavelength LED irradiation and the impact of probiotics on gut microbiota and ocular inflammation.^78^, ^79^, ^80^

Most suitable research types for this model: Mouse models are particularly suitable for mechanistic studies involving genetic manipulation, immune pathway dissection, and preliminary drug screening due to their genetic tractability and low cost.^14^, ^70^

In conclusion, mouse DED models, benefiting from genetic tractability and cost efficiency, are vital tools for mechanistic investigation and therapeutic validation. However, caution is warranted when interpreting results due to ocular anatomical and physiological differences, and findings should be corroborated with data from other animal models or clinical studies to ensure successful clinical translation.^69^, ^81^

#### 3.2.2 Rabbit models

Rabbit models offer significant advantages in DED research, primarily due to their larger eyeball size, which simplifies clinical measurements and drug administration procedures. Their ocular structure is more analogous to humans compared to small rodents, and the anatomical and physiological similarities of the lacrimal gland provide a sound experimental basis for studying DED pathology and treatments.^82^ The larger ocular surface area permits the use of standard clinical diagnostic tools such as the Schirmer test, TBUT assessment, and corneal fluorescein staining. These techniques are directly applicable in both rabbit models and patients, greatly aiding the monitoring of DED progression and the evaluation of novel therapeutics.^83^

A key limitation of rabbit models lies in the composition of their tears, which differ from those of humans. Rabbit tears contain specific lipids secreted by the Harderian gland, not typically present in human tears, potentially imparting unique influences on tear film stability.^84^ Consequently, certain DED pathological processes, particularly those involving the lipid layer in evaporative dry eye, may not be fully recapitulated. This discrepancy necessitates a careful consideration to avoid misinterpretation in translational research. Differences in corneal thickness and tear film structure between rabbits and humans may also lead to variations in drug penetration kinetics and bioavailability.^54^ Thus, despite closer physiological and anatomical resemblance, the distinct tear composition of rabbits can restrict their applicability for certain DED subtypes.

In practice, rabbit models are widely employed for drug screening and preclinical efficacy evaluation. By constructing various DED models, such as ADDE via surgical dacryoadenectomy or inflammatory models using agents like BAC, researchers can mimic diverse clinical presentations of DED.^67^, ^85^ These models effectively replicate pathological features like decreased tear film stability, corneal epithelial damage, and inflammation, and are used to assess novel therapeutics including anti‐inflammatory nano‐formulations, immunomodulators, and sustained‐release systems.^86^, ^87^ For instance, rabbit models have successfully validated the potential of anti‐inflammatory liposomes and nanocarriers in alleviating DED symptoms, aiding the transition of new drugs toward clinical application.^87^, ^88^

Most suitable research types for this model: Rabbit models are ideal for preclinical drug efficacy testing, formulation evaluation, and translational studies requiring clinical‐like ocular surface assessment tools.^89^, ^90^, ^91^

In summary, rabbit models, with their larger eyes, human‐like anatomy/physiology, and compatibility with clinical diagnostics, are pivotal in DED research. Despite limitations like tear composition differences, judicious experimental design ensures their continued irreplaceable role in probing DED mechanisms and drug screening. Their application prospects remain broad, particularly in the preclinical assessment of innovative treatment strategies.^54^, ^82^, ^84^

#### 3.2.3 Large animal models (pig, dog, non‐human primate)

Large animal models present unique advantages in DED research due to their physiological and anatomical similarities to humans, especially regarding tear protein composition and ocular structure. Pigs, as non‐primate large animals, exhibit high homology in tear proteins with humans, rendering them ideal models for translational DED studies.^91^ Comparative analyses of tear protein sequences and physicochemical properties across species identified porcine tears as having the highest similarity to humans in terms of protein profile, isoelectric point (pI), and grand average of hydropathicity (GRAVY), underscoring the high translational value of pig models for simulating human tear physiology and pathology.^92^

Dogs constitute another important large animal model. Their tear composition is similar to that of humans, and their eyeball size is comparable, facilitating the use of human clinical diagnostics and therapeutic methods for evaluation and validation. Canine DED models include both spontaneous (e.g., certain breeds prone to ADDE) and surgically induced types (e.g., lacrimal and third eyelid gland excision). These models exhibit stable, persistent dry eye symptoms, corneal neovascularization, and decreased conjunctival goblet cell function, replicating multiple pathological features of human DED.^38^, ^93^ Research on EDED and MGD in dog models is also expanding, confirming their utility for mechanistic insights and pharmacodynamic assessment.^38^

Non‐human primates (NHPs), owing to their high genomic, anatomical, and immunological similarity to humans, are paramount models for studying DED, particularly MGD. Studies show that the meibomian gland structure in NHPs like macaques resembles humans, and they develop age‐related changes analogous to human MGD, such as gland atrophy and morphological alterations, providing an ideal model foundation for investigating age‐associated DED.^94^ However, the use of NHP models is constrained by high costs, procedural complexity, and stringent ethical and regulatory hurdles.

Although large animal models offer clear benefits for translational research and efficacy verification, their limitations are substantial. Husbandry and maintenance expenses far exceed those for small rodents, and surgical procedures and long‐term monitoring are more complex, increasing research demands. Studies involving dogs and NHPs also face rigorous ethical scrutiny, limiting their widespread adoption. Researchers must therefore carefully balance biological relevance against cost and ethical considerations when selecting the most appropriate model.

Most suitable research types for this model: Large animal models (especially non‐human primates) are best suited for translational validation, pharmacokinetic (PK) studies, and complex pathophysiology investigations where close anatomical and physiological resemblance to humans is critical.^62^, ^94^

In conclusion, pigs, dogs, and non‐human primates, as large animal models, provide significant advantages through their high similarity to human tear proteins and ocular anatomy, making them well suited for pathological mechanism investigation and drug efficacy validation. With ongoing advancements in gene editing and imaging diagnostics, these models are poised to assume greater roles in translational medicine, accelerating the development and clinical application of new DED therapies.^38^, ^92^, ^93^

### 3.3 Investigating inflammatory mechanisms in dry eye animal models

**FIGURE 4:**
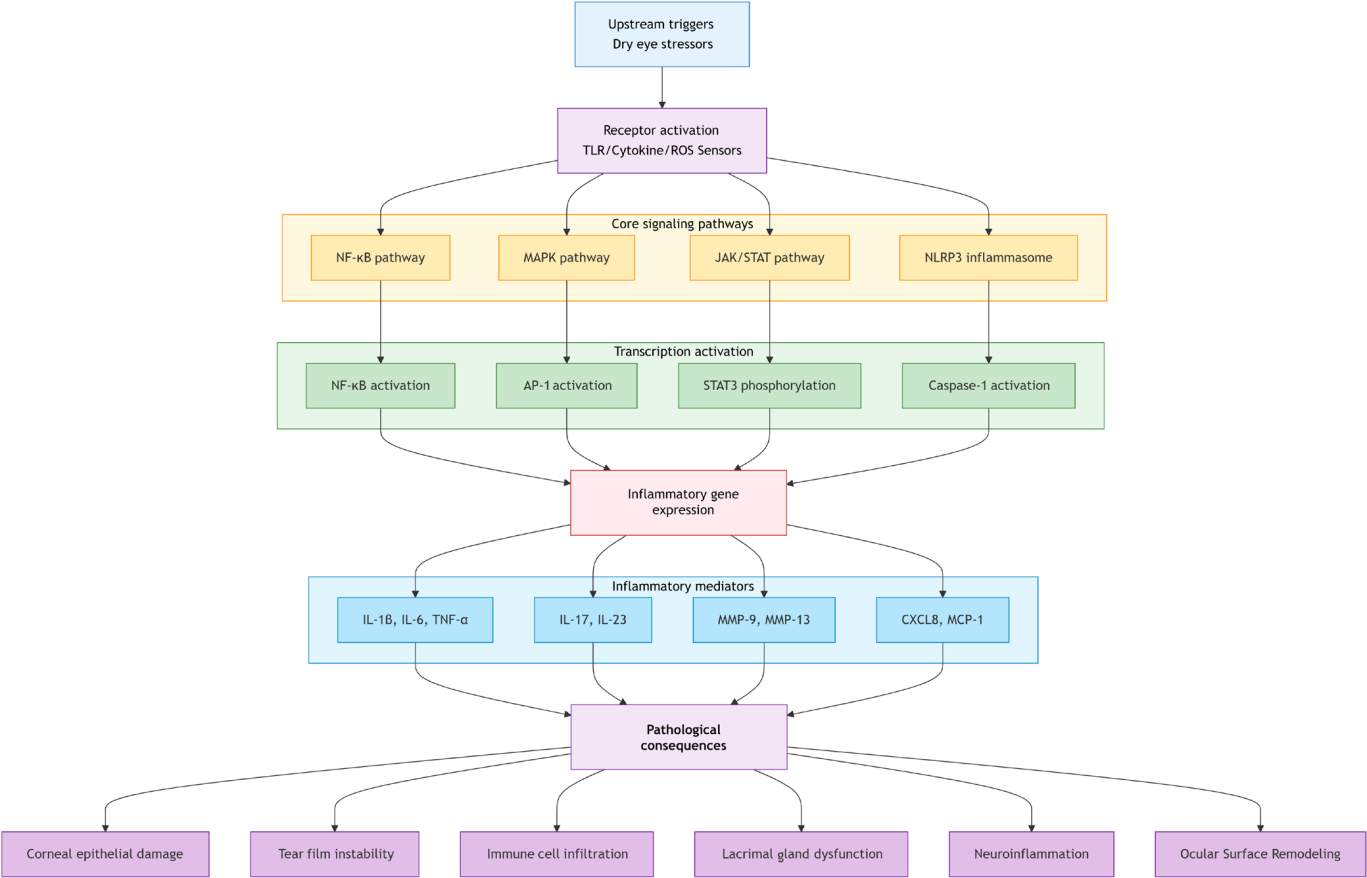
Simplified schematic of core inflammatory signaling pathways in DED pathogenesis.

Figure 4 depicts a multitiered network mechanism, systematically illustrating the cascade from upstream triggers to core pathway activation, downstream release of inflammatory effectors, and the resultant pathological consequences, providing a clear framework for understanding DED‐associated inflammation. The diagram outlines key pathways from initial stress to pathological outcomes, highlighting major nodes (e.g., NF‐κB, NLRP3) and effector molecules (e.g., IL‐1β).

#### 3.3.1 Inflammatory signaling pathway activation

Inflammatory responses are integral to DED pathogenesis, contributing to tear film destabilization and corneal injury via multiple signaling cascades. Animal model research highlights the critical regulatory role of the NF‐κB signaling pathway in DED‐related inflammation.^95^ Specifically, NF‐κB and its downstream pro‐inflammatory cytokines, notably IL‐1β and TNF‐α, are significantly upregulated, concomitant with inflammatory activation and damage in corneal epithelial cells.^96^ Studies utilizing BAC‐ or scopolamine‐induced dry eye models report marked elevations of IL‐1β, TNF‐α, and other inflammatory mediators in corneal and lacrimal gland tissues, underscoring their contribution to tear film instability and ocular surface damage.^33^, ^97^

As a canonical inflammatory signal transducer, NF‐κB, upon activation in DED models, promotes the transcription and secretion of numerous inflammatory factors, creating an amplification loop that further disrupts corneal epithelial integrity and tear film stability. In dry eye models, miR‐146a negatively regulates inflammation via the IRAK1/TRAF6/NF‐κB axis, indicating a close relationship between pathway activity and inflammatory severity.^98^ Moreover, the hyperosmolar stress inherent to the dry eye state activates various inflammatory pathways, including MAPK and JAK/STAT3, exacerbating corneal inflammation and fibrotic processes.^99^, ^100^ Notably, sympathetic nervous system activation, via the norepinephrine‐β2 adrenergic receptor pathway, promotes NF‐κB activation, driving DED pathogenesis and revealing crosstalk between neuroinflammatory and classical inflammatory signaling.^26^

Animal studies demonstrate significantly elevated levels of inflammatory cytokines like IL‐1β, IL‐6, and TNF‐α in tears following DED induction, with their expression levels correlating positively with the extent of corneal epithelial damage.^97^ Persistent inflammation also induces alterations in corneal nerve morphology and function, further aggravating ocular surface injury.^101^, ^102^ Interventions targeting inflammatory signaling, such as anti‐inflammatory drugs delivered via nano‐carriers (e.g., tetrahydroflavone nano‐formulations), effectively suppress NF‐κB pathway activity, mitigating corneal epithelial damage and inflammation,^88^ highlighting the therapeutic potential of modulating these pathways.

In summary, the upregulation of NF‐κB, IL‐1β, TNF‐α, and other inflammatory factors in animal models typifies inflammatory pathway activation in DED. These mediators engage multiple signaling routes to participate in the pathology of tear film instability and corneal damage. Delving into the activation mechanisms and interactions of these pathways is crucial for unraveling DED pathology and providing a rationale for targeted anti‐inflammatory therapies. Future investigations should further explore the interplay between inflammatory signaling, neural regulation, and immune cell activation to inform the design of integrated DED treatment strategies.^9^, ^32^, ^103^

#### 3.3.2 Immune cell infiltration and regulation

Immune cells are pivotal players in DED pathogenesis, particularly within the inflammatory milieu of the lacrimal gland and cornea. Significant infiltration of lymphocytes, macrophages, and other immune cells into lacrimal gland tissue is observed, strongly associated with SS‐related DED.^96^ Research using NOD mouse models reveals that progressing SS‐related DED is accompanied by elevated DNA methylation levels in the lacrimal gland, with notable methylation alterations in genes governing T‐cell activation, proliferation, and differentiation (e.g., Itgal, Vav1, Irf4, Icosl).^104^ The increased expression of these genes positively correlates with the degree of CD4+ T‐cell infiltration in the lacrimal gland, implicating T‐cell‐mediated immunity in DED inflammation.^105^ Immune cell infiltration is not confined to the lacrimal gland; the immune cell composition of the cornea and conjunctiva also undergoes significant changes. In Aire‐deficient mouse models, early dry eye lesions feature moderate immune cell infiltration, prominently involving macrophages and dendritic cells, leading to altered corneal biomechanics and disruption of the epithelial basement membrane, thereby worsening corneal inflammation and neural damage.^106^

Autoimmune models like the NOD.H‐2h4 double‐knockout mouse further clarify SS‐related immune mechanisms. This model shows age‐dependent worsening of corneal damage, earlier onset in females, B‐cell predominance in the lacrimal gland alongside helper T cells and macrophages, and lacrimal gland inflammation linked to heightened P2X7 and P2Y2 receptor expression, indicating that mediator‐immune cell interactions fuel DED inflammation.^53^ The S100A8/A9 complex is significantly upregulated in SS‐DED, promoting dendritic cell‐driven Th17 responses and enhancing pro‐inflammatory cytokine expression via the Acod1/STAT3 pathway, unveiling a new target for DED immunomodulation.^107^

Within the DED immune environment, the inflammatory mediator IFN‐γ directly promotes inflammatory responses in corneal stromal fibroblasts and epithelial cells, inducing cytotoxicity and barrier dysfunction, thereby aggravating clinical manifestations.^108^ Conjunctival goblet cell loss closely associates with increased infiltration of monocytes and dendritic cells, highlighting the significance of immune‐goblet cell interactions for ocular surface homeostasis.^109^ Recent studies also report markedly increased diversity of infiltrating immune cells in the eye, encompassing neutrophils, dendritic cells, macrophages, and various T‐cell subsets (CD4+, CD8+, Th17), alongside reduced regulatory T‐cell numbers. These immune shifts correlate with DED clinical signs like corneal staining and TBUT.^110^

Immune regulation in DED extends beyond cell infiltration and activation to include complex networks of epigenetic control and cytokine signaling. DNA methylation regulates T‐cell‐related gene expression, the IL‐33/ST2 axis mediates immune responses, and multiple pathways (e.g., MAPK, TNF, IL‐17) participate in inflammation.^15^, ^105^, ^111^ These findings underscore that immune cells in DED not only act as inflammatory effectors but also, via cytokine secretion and intercellular signaling, help establish a self‐sustaining pro‐inflammatory microenvironment that propels disease progression.

In conclusion, the infiltration and regulatory dynamics of immune cells in the lacrimal gland and cornea form a critical pathological basis for DED, particularly its autoimmune forms. In‐depth investigation of immune cell types, activation states, and secretory profiles, combined with insights from autoimmune models, provides a theoretical foundation and identifies potential targets for immunomodulatory therapies, fostering innovation in DED diagnosis and treatment.

#### 3.3.3 Validation of inflammation‐related therapeutic targets

Given the centrality of inflammation in DED pathogenesis—causing tissue damage, exacerbating tear dysfunction, and perpetuating a vicious cycle of symptom worsening—therapeutic strategies targeting inflammation are a major focus of current DED research and clinical practice. Animal models are indispensable for validating the efficacy of anti‐inflammatory drugs and novel interventions and crucial for elucidating mechanisms, assessing potential, and enabling clinical translation.

Animal models have proven essential for evaluating the efficacy of anti‐inflammatory agents. Conventional drugs like cyclosporine A have been demonstrated in various dry eye models to significantly reduce inflammation and improve ocular surface health.^112^ Clinical and animal studies show that cyclosporine A combined with punctal occlusion is more effective than either alone in enhancing tear secretion and reducing levels of inflammatory factors such as IL‐6, IL‐1, and MMP‐9, thereby alleviating ocular surface inflammation.^90^ Advances in nano‐technology have also been applied in rabbit models; for example, tetrahydroflavone‐loaded nanoliposomes effectively suppressed DED‐associated inflammation without significantly affecting intraocular pressure, demonstrating favorable biosafety and anti‐inflammatory activity.^86^

Stem cells and their derivatives, particularly MSCs and their exosomes, have emerged as a prominent research area. MSCs and their exosomes modulate immune‐inflammatory responses and promote tissue repair through multiple mechanisms, showing significant anti‐inflammatory and tissue‐protective effects in DED mouse models. Human umbilical cord MSC‐derived exosomes (HUCMSC‐EXO) mitigate corneal epithelial cell apoptosis and inflammation by targeting SQSTM1 gene expression via miR‐146a, substantially improving DED symptoms.^75^ Bone marrow MSCs alleviate oxidative stress and inflammation, promote corneal epithelial proliferation, reduce inflammatory damage, and ameliorate DED by inhibiting the ROS‐NLRP3‐IL‐1β signaling axis.^113^ Such cell‐based strategies open new avenues for DED treatment.

Traditional therapies like acupuncture and electroacupuncture are also widely investigated for inflammation modulation in DED. Studies in New Zealand white rabbits and mice indicate that acupuncture/electroacupuncture reduce corneal and lacrimal gland expression of IL‐1, TNF‐α, and IL‐8 by modulating the α7 nicotinic acetylcholine receptor (α7nAChR) and NF‐κB pathway, promoting tear secretion and ocular surface repair, with anti‐inflammatory effects rivaling steroids.^114^, ^115^, ^116^ These findings provide a molecular basis for acupuncture's mechanism and support its role as an adjunct therapy.

Targeted inhibitors against specific inflammatory pathways also show promise in animal models. Macrophage‐associated inflammation mediated by integrin αv and its ligand vitronectin was identified as a significant DED mechanism. An αv integrin inhibitor markedly reduced corneal damage and inflammation in a mouse DED model.^117^ Similarly, ICI 118551, a selective β2‐adrenergic receptor (β2‐AR) blocker, improved tear secretion dysfunction and corneal inflammation by inhibiting NF‐κB and IL‐17 signaling in a mouse model, revealing a novel therapeutic target.^26^

Antioxidants represent another important direction. Astaxanthin protected corneal epithelial cells by activating the Keap1‐Nrf2/HO‐1 antioxidant pathway, reducing inflammation and oxidative damage in a BAC‐induced mouse DED model.^118^ Nanozyme‐based eye drops interrupted DED's inflammatory cycle by scavenging excess reactive oxygen species (ROS) and inhibiting NLRP3 inflammasome activation.^119^

Exogenous supplementation of proteins like the lubricating factor PRG4 also exhibits anti‐inflammatory properties. PRG4, beyond its lubricating function, inhibits inflammatory factors and MMP‐9 activity. Its expression is decreased in DED mouse models, and its supplementation helps alleviate inflammation.^120^

In summary, animal models have been instrumental in exploring DED inflammatory mechanisms and validating anti‐inflammatory strategies. Utilizing genetically modified mice, drug‐induced models, and various species, researchers can simulate different DED subtypes and systematically assess the efficacy and safety of anti‐inflammatory drugs, stem cells/exosomes, traditional treatments, and novel targeted therapies. Future combination therapies addressing multiple targets and mechanisms hold promise for overcoming the limitations of single‐target approaches, offering more effective anti‐inflammatory treatments for DED patients and advancing the field toward precision medicine.^9^, ^32^, ^70^, ^82^

#### 3.3.4 Summary and future perspectives in inflammatory mechanism research

Current consensus indicates that inflammation is a central driver of DED pathogenesis, involving the activation of key signaling pathways (e.g., NF‐κB, Mitogen‐Activated Protein Kinase (MAPK), Janus Kinases (JAK)/Signal Transducer and Activator of Transcription (STAT)), immune cell infiltration, and cytokine dysregulation.^15^ However, several key issues remain unresolved: (1) the similarities and differences in inflammatory features among different DED subtypes (e.g., ADDE vs. EDED, autoimmune vs. non‐autoimmune), (2) the specific mechanisms of neuro‐immune interactions in sustaining ocular surface inflammation, and (3) the temporal dynamics of inflammatory responses during disease progression and treatment.^121^ Future technological breakthroughs, such as single‐cell RNA sequencing and spatial transcriptomics applied to DED animal models, hold promise for unraveling immune cell heterogeneity, mapping inflammatory networks, and identifying novel therapeutic targets.^122^

### 3.4 Animal model research on neural injury and pain mechanisms

#### 3.4.1 Neuroinflammation and pain perception

DED frequently involves neuropathic pain and neuroinflammatory components alongside tear deficiency and surface damage.^123^ The complex pathophysiology of pain perception involves various neurotransmitters and ion channels, with Substance P (SP) and Transient Receptor Potential Vanilloid 1 (TRPV1) pathways being particularly prominent in dry eye models. SP, a neuropeptide abundant in trigeminal ganglia and corneal nerve endings, participates in inflammation and pain signaling. Its expression increases in DED, promoting inflammatory mediator release, nerve ending sensitization, and heightened pain perception.^124^ TRPV1, a key ion channel in nociception responsive to heat, acid, and chemicals, also contributes significantly to DED‐related neuropathic pain. TRPV1 upregulation in dry eye models exacerbates corneal nerve sensitivity and pain responses. Topical application of the TRPV1 antagonist capsazepine effectively alleviated corneal pain and neuroinflammation, reducing related gene expression and demonstrating analgesic potential.^124^

The P2X3 receptor, another pain‐related channel, is also implicated in DED‐induced neuropathic pain. Electroacupuncture treatment reduced neuropathic pain and inflammation in dry eye animal models by suppressing the P2X3R and Protein Kinase C (PKC) signaling pathway, confirming its role in neuroinflammation.^125^, ^126^ This mechanism underpins the development of P2X3R‐targeting neuroprotective agents for DED pain management. The NK1 receptor, SP's primary receptor, also showed analgesic effects in dry eye models upon antagonism, suggesting that blocking the SP‐NK1R axis can relieve DED‐associated pain.^127^

Abnormal pain perception in DED patients is closely linked to neuroinflammation and often co‐occurs with psychological comorbidities like anxiety and depression, complicating the disease and its management. Neuroprotective agents and NK1R antagonists may provide analgesia and improve quality of life by modulating neuro‐immune interactions and reducing nerve ending hyperexcitability and inflammation.^128^, ^129^ In conclusion, SP and TRPV1 pathways contribute not only to pain signaling but also to neuroinflammation development in DED. Therapeutic strategies targeting these pathways offer new directions for managing DED‐related neuropathic pain.

#### 3.4.2 Corneal nerve morphological changes

The cornea, one of the most densely innervated tissues, plays a key role in DED pathogenesis through structural neural alterations. Immunofluorescence and IVCM are powerful techniques for visualizing corneal nerve microstructure. Studies using these methods consistently show reduced corneal nerve density and significant morphological changes in DED patients.^130^, ^131^

IVCM and immunofluorescence studies reveal that the corneal subbasal nerve plexus in DED patients exhibits decreased NFL and NFD, alongside alterations in nerve branch density (NBD). Some studies report reduced NFL and NFD but increased NBD, possibly reflecting nerve damage and aberrant regeneration.^130^, ^131^, ^132^ Increased nerve tortuosity and reflectivity also suggest morphological disarray. The presence of immune cells like dendritic cells can confound nerve quantification; some studies improve accuracy by digitally removing their interference.^133^, ^134^

Corneal nerve morphological changes correlate with DED symptoms. Reduced nerve density and altered morphology often accompany decreased corneal sensitivity, contributing not only to abnormal tear secretion but also to neuropathic symptoms like pain and dysesthesia. A systematic review indicates generally reduced mechanical corneal sensitivity in DED patients, supporting impaired nerve function despite methodological variations.^135^ Nerve morphology also correlates with pain perception; for instance, NBD may negatively correlate with TBUT, linking structural changes to tear film stability.^131^, ^136^

Immunofluorescence studies further connect nerve damage to pain symptoms. Corneal nerve terminal injury can incite neuroinflammation, activating peripheral and central nervous systems and fostering chronic pain. Dry eye models, especially under inflammatory conditions, show altered spatial distribution of pain‐related neuropeptides like calcitonin gene‐related peptide (CGRP) in nerve fibers, aberrant nerve regeneration, and associated pain perception changes.^137^, ^138^ Nerve injury‐induced sensory abnormalities positively correlate with anxiety and pain scores in DED patients, underscoring the role of nerve morphology in DED pain mechanisms.^126^, ^139^

In summary, immunofluorescence and IVCM have unequivocally identified corneal nerve morphological changes—reduced density, abnormal fiber morphology, altered branching—in DED. These structural deficits correlate with functional impairment and DED‐related pain. Future research employing advanced image analysis, functional assessment, and molecular biology will further clarify the mechanisms of corneal nerve damage in DED, informing neuroprotective and regenerative strategies.^130^, ^131^, ^135^, ^137^

**FIGURE 5:**
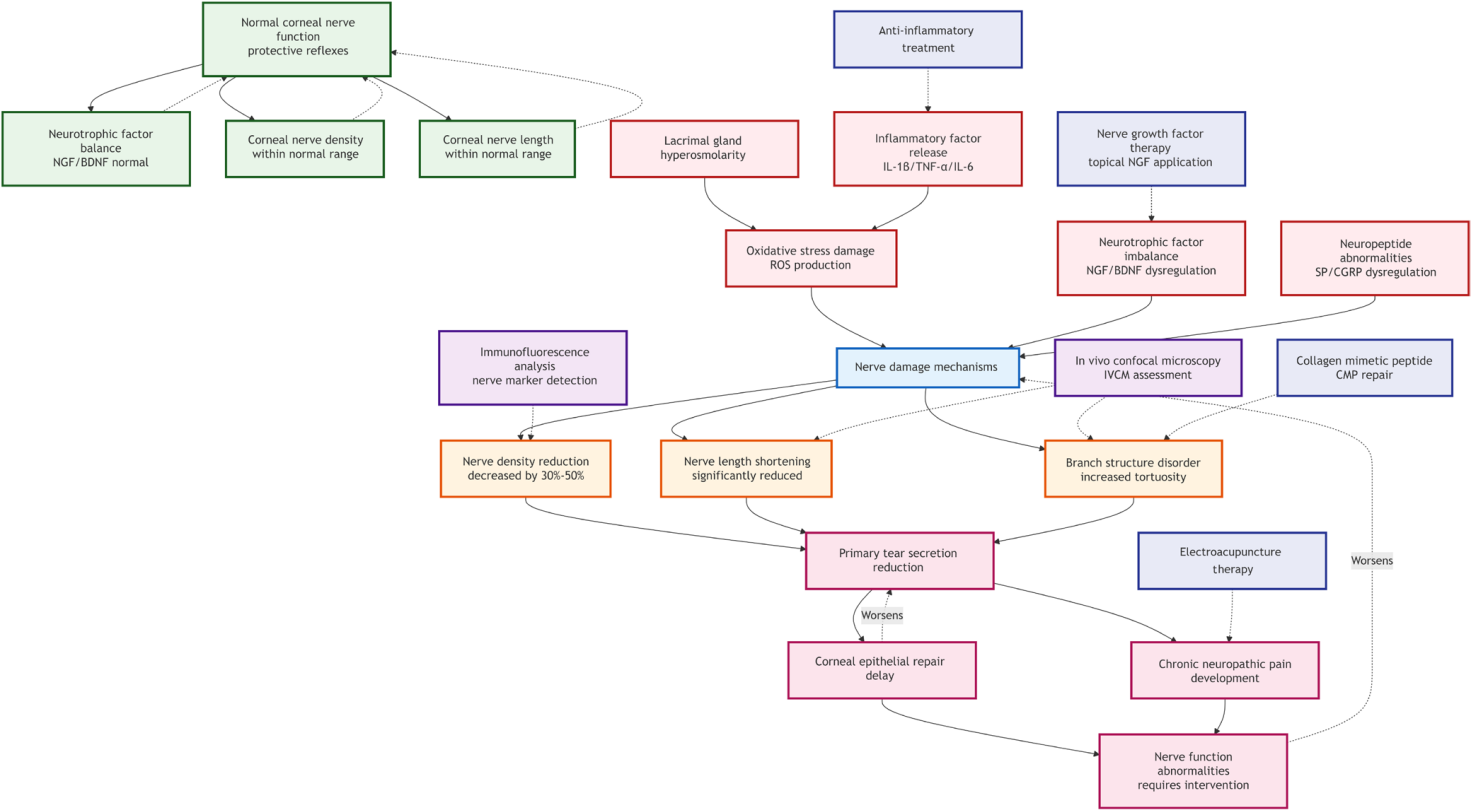
Network mechanism diagram of corneal nerve morphological changes.

Figure 5 presents a multi‐node network diagram systematically outlining the complex mechanisms of corneal neuropathy in DED. It clearly illustrates the progression from normal nerve characteristics to pathological factor involvement, subsequent structural/functional neural changes, and the resulting downstream pathological effects. The diagram also incorporates assessment methods and therapeutic intervention strategies, offering a comprehensive perspective on DED‐associated neural pathology.

#### 3.4.3 Nerve repair and regeneration strategies

Nerve repair and regeneration represent a critical frontier in DED therapeutics, aiming to address the functional deficits and symptoms stemming from ocular surface nerve injury. The ocular surface, particularly the cornea, is richly innervated by sensory nerves that not only transmit sensations but also regulate tear secretion, promote epithelial repair, and maintain tissue homeostasis. DED and related conditions often involve morphological and functional corneal nerve abnormalities—thinning fibers, decreased density, impaired conduction—exacerbating ocular surface inflammation and tear deficiency in a vicious cycle.^101^

Pharmacological agents and stem cell therapies promoting nerve regeneration are current research foci. Specific fatty acid‐derived signaling molecules, like Neuroprotectin D1 (NPD1) and the novel Resolvin D6 isomer (RvD6i), significantly promote the regeneration of injured corneal nerves, improve wound healing and tear secretion, and concurrently alleviate neuropathic pain. This primarily involves activating calcium‐independent phospholipase A2, releasing membrane‐embedded fatty acids, and enhancing the synthesis of neurotrophic factors like brain‐derived neurotrophic factor (BDNF), nerve growth factor (NGF), and Semaphorin 7A, fostering neurogenesis and functional recovery.^140^ Other factors, like basic fibroblast growth factor (bFGF), also promote corneal nerve regeneration and partially restore tear secretion in animal models, suggesting new therapeutic avenues.^141^

Stem cell therapies, particularly using placental‐derived products like amniotic membrane and umbilical cord serum (UCS), show considerable promise. Amniotic membrane transplantation offers anti‐inflammatory and anti‐fibrotic benefits and promotes corneal epithelial repair and nerve regeneration via its rich cocktail of growth factors and cytokines. UCS contains numerous neurotrophic factors and anti‐inflammatory components that support nerve repair. It has been used for various ocular surface disorders, including DED and neurotrophic keratitis, demonstrating potential for symptom relief and corneal nerve recovery.^142^ These biologicals not only aid nerve regeneration but also modulate the immune microenvironment, mitigating post‐injury inflammation and supporting overall neural functional restoration.

Animal models are irreplaceable for studying nerve repair mechanisms. Mouse models of corneal sensory nerve injury reveal that damage affects not only local nerve structure but can also disrupt lacrimal gland function and reduce tear secretion via central influences (e.g., on the superior salivatory nucleus), accompanied by phase delays in circadian clock gene expression and downregulation of immune signaling pathways. These findings unveil the complex neuro‐immune‐endocrine interactions triggered by nerve injury, suggesting new targets for DED nerve repair strategies. Interventions like exogenous bFGF can partially restore tear secretion, indicating the potential value of nerve repair therapies.^141^

In conclusion, DED animal models provide a vital platform for exploring nerve repair mechanisms and advancing nerve regenerative pharmacotherapy and cell‐based therapies. Future work should clarify the mechanisms of different neurotrophic factors and regeneration strategies, optimize combination therapies, and evaluate long‐term safety and efficacy to drive clinical translation. Integrating diverse approaches—fatty acid‐derived signals, placental derivatives, growth factors—holds promise for breakthroughs in DED nerve repair/regeneration, ultimately improving patients' quality of life.^140^, ^141^, ^142^

### 3.5 Evaluation indicators and diagnostic methods for dry eye animal models

#### 3.5.1 Clinical manifestation assessment

Clinical evaluation is a cornerstone of DED animal model research, employing objective parameters to gauge pathophysiological changes and disease severity. Key indicators include corneal fluorescein staining, TBUT, tear volume measurement, ocular surface inflammation scores, and conjunctival goblet cell counts.

Corneal fluorescein staining assesses epithelial damage. Decreased tear film stability in DED models damages the corneal epithelium, increasing the area and intensity of fluorescein uptake. For example, rabbit MGD models established via chemical or electrocautery methods show significantly elevated fluorescein scores, indicating tear film disruption and epithelial compromise.^143^ BAC‐induced models also exhibit exacerbated corneal damage and inflammation, reflected by expanded staining areas.^89^, ^118^

TBUT measures tear film stability. DED patients and animal models consistently demonstrate shortened TBUT, indicating increased tear film fragility. In rabbit MGD models, non‐invasive TBUT (NITBUT) decreased significantly by day 30 post‐induction and remained reduced for 90 days, confirming persistent instability.^143^ Similarly, BAC and desiccated stress‐induced mouse models show shortened TBUT, validating its consistency across different DED models.^144^, ^145^

Tear volume measurement, often via phenol red thread test or Schirmer's test, evaluates lacrimal gland function, particularly in aqueous‐deficient models. Models using hydroxypropyl methylcellulose or scopolamine show markedly reduced tear secretion, indicating impaired lacrimal function. The scopolamine‐induced rat model demonstrates significant tear reduction alongside corneal/conjunctival pathology, making it ideal for studying lacrimal deficiency.^24^, ^33^ Desiccating stress model mice also exhibit low tear volume, characteristic of aqueous‐deficient DED.^145^

Ocular surface inflammation scoring (corneal/conjunctival staining) quantifies mucosal damage and inflammation severity. Increased inflammatory cell infiltration and cytokine levels correlate with higher scores in both clinics and models.^58^, ^90^ The BAC model shows elevated inflammatory cytokines (IL‐1β, IL‐6, MMP‐9) concurrent with worse staining scores.^89^ Combination therapy with cyclosporine and punctal plugs significantly lowered inflammation scores and cytokine levels, improving ocular surface health.^90^

Conjunctival goblet cell count reflects the secretory and protective capacity of the conjunctival epithelium. Goblet cell loss is a common DED feature across animal models. Desiccating stress mice show significantly decreased goblet cell density, indicating impaired mucus secretion.^145^ Conversely, cyclosporine+punctal plug treatment in rabbit models increased goblet cell numbers, demonstrating therapeutic benefit for ocular surface protection.^90^ As crucial components of the tear film, goblet cell counts are vital for assessing pathology and treatment response.

In summary, clinical assessment of DED animal models utilizes a multi‐parameter approach—fluorescein staining, TBUT, tear volume, inflammation scores, goblet cell count—to comprehensively evaluate tear film stability, secretion, and ocular surface inflammation. Standardized, objective application of these methods ensures accurate model validation and provides a solid foundation for mechanistic studies and therapeutic screening.^24^, ^90^, ^143^, ^145^

#### 3.5.2 Molecular biology indicators

Molecular biology indicators are crucial for evaluating pathological changes and mechanisms in DED animal models, focusing primarily on inflammatory factor expression, corneal epithelial marker alterations, and related gene expression analyzed via immunohistochemistry (IHC) and polymerase chain reaction (PCR).

Inflammation being central to DED, related cytokines, and chemokines are typically upregulated in animal models, driving tissue damage. Pro‐inflammatory factors like IL‐1β, TNF‐α, and IL‐6 are significantly elevated, correlating with corneal epithelial structural and functional impairment.^32^, ^144^ Corneal epithelial‐specific markers, such as keratin K12 and transcription factor Pax6, are often downregulated in dry eye models, indicating abnormal differentiation and compromised regeneration. Lacrimal gland excision models show markedly reduced K12 and Pax6 expression alongside irregular epithelial hyperplasia and inflammation.^23^

IHC localizes and visualizes protein expression. In lacrimal gland excision mice, immunofluorescence shows decreased K12 and Pax6 with concurrent increased Ki67 (proliferation marker), suggesting aberrant epithelial proliferation.^23^ IHC also detects immune cell infiltration (e.g., CD4+, CD8+ T cell distribution), revealing ocular surface immune landscape shifts.^146^ Furthermore, IHC can visualize nerve damage markers, showing reduced expression in DED models, reflecting neuropathy.^144^, ^147^

PCR, especially quantitative real‐time PCR (qPCR), quantifies gene expression. Analyzing transcripts of inflammatory mediators (IL‐1β, TNF‐α, IL‐6, MMP‐9) and immunoregulators quantifies the inflammatory response's intensity and duration.^32^, ^144^ PCR also confirms the downregulation of corneal epithelial genes (K12, Pax6) and upregulation of abnormal differentiation genes (e.g., Sprr1b), supporting molecular mechanisms of epithelial damage/repair failure.^23^ Additionally, PCR analyzes immune cell‐related gene expression, such as T‐cell receptor (TCR) gene rearrangement and clonality, revealing dynamics of immune activation and memory T cells in DED models, providing a molecular basis for immune mechanisms.^81^

In summary, molecular biology indicators—inflammatory factors, corneal epithelial markers—reflect key DED pathological processes. Combining IHC and PCR provides qualitative/quantitative pathological data and unveils molecular mechanisms and immune regulation dynamics. These indicators offer a scientific basis for DED pathology research and novel therapy evaluation, driving mechanistic understanding and clinical translation.^23^, ^32^, ^81^, ^144^

#### 3.5.3 Multi‐parameter comprehensive analysis methods

Diagnosing and assessing DED in animal models often relies on multiple clinical and physiological parameters (tear volume, TBUT, corneal staining, inflammation markers). Given DED's complex, multifactorial nature, single indicators are insufficient to capture the full disease state. Multi‐parameter comprehensive analysis methods thus enhance diagnostic accuracy and research rigor. Principal component analysis (PCA), a classic dimensionality‐reduction technique, integrates multiple correlated variables into fewer, representative principal components, simplifying data structure and revealing underlying variation patterns.

Applied to DED animal models, PCA successfully extracted the first principal component (PC1) from parameters like TBUT, tear osmolarity, Schirmer test, and corneal staining scores. PC1 comprehensively represented DED pathological status, effectively distinguishing healthy from dry eye groups and explaining 73% of data variability, outperforming any single metric.^148^ PCA also visually displays inter‐group similarities/differences via 2D/3D plots, offering new tools for disease state grading/classification.^148^

Multi‐parameter analysis improves diagnostic sensitivity/specificity and provides a more holistic drug efficacy evaluation framework. Traditional assessment often focuses on single metric improvement, potentially missing broader therapeutic impacts. Methods like PCA integrate multiple clinical/molecular indicators into a composite score, objectively evaluating overall drug efficacy and its effects on different pathological aspects. For instance, comparing inflammation, neural injury, and tear film stability across different models (lacrimal excision, BAC, desiccation) using comprehensive analysis helps identify model similarities/differences, guiding optimal model selection for specific research questions.^12^, ^144^

Furthermore, multi‐parameter analysis promotes model standardization and quantification. Principal component‐based scoring systems enable more precise DED severity grading (mild, moderate, severe), bridging translational clinical pathology research. Such methods also reduce inter‐individual/experimental variation, enhancing result reproducibility/reliability. Integrating advanced data analysis like machine learning (ML) and multi‐parameter comprehensive analysis will play an expanding role in phenotyping, mechanism investigation, and drug screening using DED animal models, propelling the field toward precision medicine.^13^, ^148^

**FIGURE 6:**
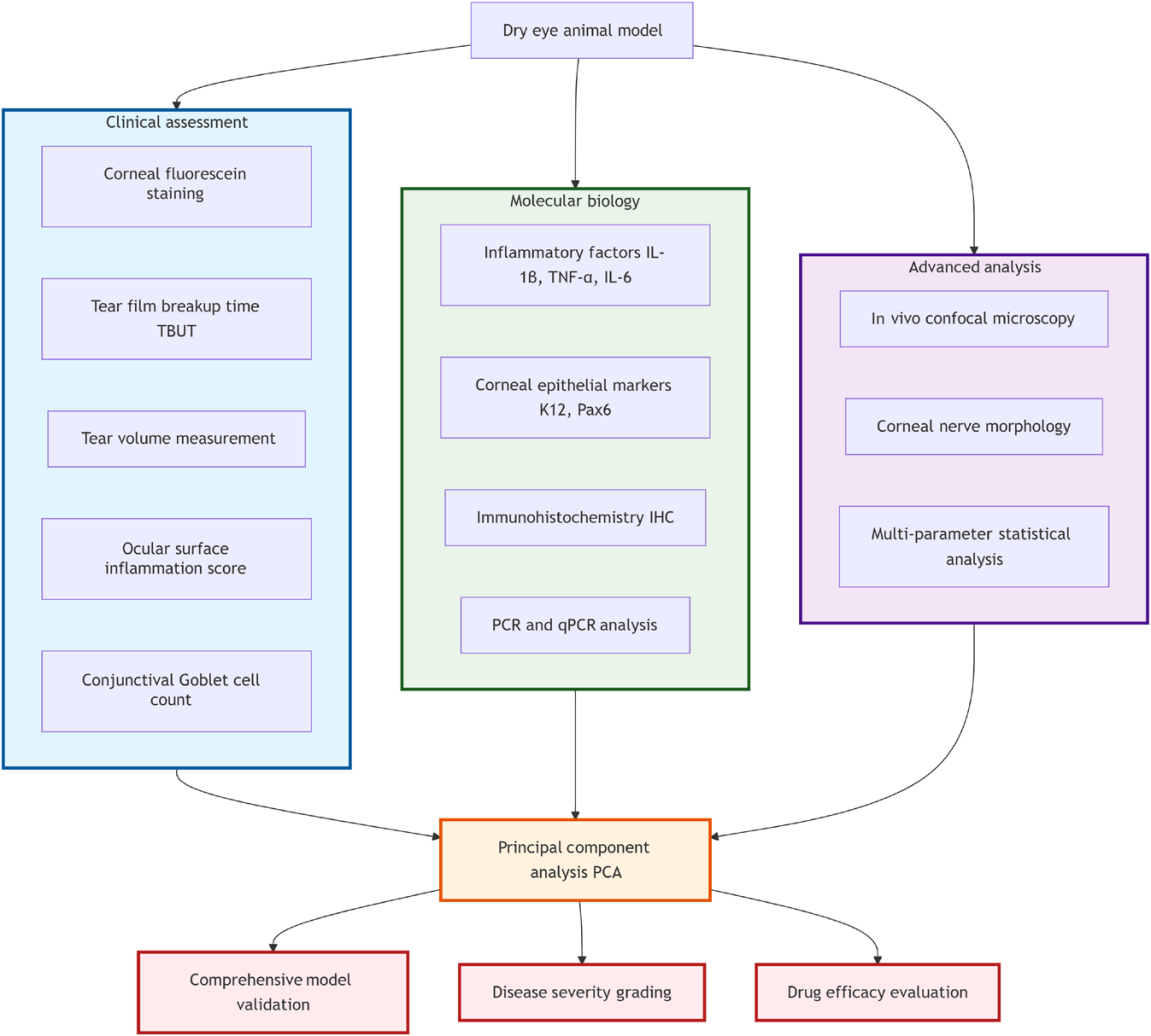
Multi‐parameter comprehensive evaluation system.

Figure 6 uses a flowchart to depict the modern evaluation paradigm for DED animal models, emphasizing a multilevel, integrated assessment system spanning macroscopic clinical manifestations, microscopic molecular mechanisms, and advanced structural/functional analyses, culminating in objective, accurate model validation through statistical integration.

#### 3.5.4 Translational gaps and model optimization strategies

Despite their utility, existing DED animal models often fail to fully recapitulate key clinical complexities. Heterogeneity: Clinical DED presents as a spectrum, whereas most models represent a single subtype.^149^ Comorbidities: Common clinical associations (e.g., DED in rheumatoid arthritis, diabetes) are rarely modeled.^150^ Aging: Most models use young adult animals, whereas DED prevalence increases with age.^151^ To bridge these gaps, future models should (1) incorporate aging factors (e.g., aged animals or progeroid models), (2) develop combinatorial models simulating comorbidities (e.g., DED + diabetic neuropathy), and (3) integrate longitudinal monitoring to capture disease progression variability. Such “next‐generation” models will enhance clinical predictive value.

### 3.6 Applications and future directions of dry eye animal models

#### 3.6.1 New drug screening and treatment strategy evaluation

Dry eye animal models are vital for screening new therapeutics and evaluating treatment strategies. First, they simulate diverse DED pathologies—inflammation, neural damage, tear deficiency—enabling efficacy testing of anti‐inflammatory drugs, neuroprotective agents, and biologics. The BAC‐induced model, for example, exhibits significant ocular surface damage and inflammation, particularly enhanced Th17 responses, providing targets for anti‐inflammatory mechanism studies.^144^ The LGE model shows pronounced corneal nerve injury, suitable for assessing neuroprotectants.^144^ These models allow precise control over drug administration and dosage, facilitating systematic efficacy/safety evaluation. Cytisine (CYT) eye drops promoted cell migration, reduced oxidative stress, and inhibited inflammatory signaling in the BAC‐induced mouse model, demonstrating promising efficacy and safety.^152^ The tyrosine kinase inhibitor imatinib mesylate effectively reduced ocular surface damage and immune cell infiltration in chemical DED models, outperforming cyclosporine and suggesting its potential as a novel ophthalmic drug.^89^

Second, integrating model‐derived clinical and molecular data optimizes treatment design. DED clinical signs—reduced tear volume, shortened TBUT, corneal damage—have corresponding assessments in models. Advanced corneal imaging (e.g., Oculus Keratograph 5M) dynamically monitors tear film stability and corneal irregularity in rabbit models, providing objective drug efficacy data.^83^ Measuring ocular surface/tear inflammatory factors (IL‐1β, IL‐6, MMP‐9) reflects drug‐induced immunomodulation.^28^, ^90^ Preclinically, combining these indicators allows multidimensional efficacy assessment. Cyclosporine + biodegradable punctal plugs improved tear secretion, tear film stability, and reduced multiple inflammatory factors in patients and models, demonstrating the superiority of combination therapy.^90^ Natural products/derivatives like SM934 and olive pomace polyphenols showed significant immunomodulatory/anti‐inflammatory effects in models, supporting novel biologic development.^153^, ^154^

In summary, DED animal models provide reliable platforms for drug screening. Combined with clinical and biomarker data, they enable precise design/optimization of treatment strategies, shifting DED management from symptomatic relief to multitarget, multi‐mechanism intervention, offering scientific/technical support for clinical practice.^89^, ^90^, ^144^ Future use should include evaluating emerging biomaterials/drug carriers (nanomedicines, hydrogels) to enhance ocular surface bioavailability/duration, fostering DED treatment innovation.^155^

#### 3.6.2 Model optimization and diversification

DED animal model development is evolving toward greater human pathological fidelity and multifactor interaction to enhance clinical relevance. NHP model development is a key focus. NHPs' ocular anatomy, physiology, and immunity closely resemble humans, making their DED models highly translatable for pathology and drug response. Environmentally stressed NHP models using controlled temperature/humidity/airflow closely mimic human DED clinical manifestations and treatment response, serving as important platforms for novel drug evaluation.^62^ However, high costs and ethical restrictions limit NHP use, requiring further refinement/standardization.

Model diversification involves combining environmental, immune, and neural pathogenic mechanisms into multifactorial composites. Models combining BAC‐induced inflammation, lacrimal dysfunction, and neural injury successfully simulate DED inflammatory/neuropathic features.^67^, ^144^ Such models better reflect DED complexity, revealing dynamic interactions between different factors. Animal studies combining immunosuppressants (e.g., cyclosporine) with physical occlusion (punctal plugs) showed enhanced symptom/inflammation improvement, guiding treatment optimization.^90^

DED immune cell diversity/dynamics are also incorporated. Flow cytometry and TCR sequencing revealed extensive ocular surface immune cell diversity and specific clonal expansion in DED, enabling models that reflect immune microenvironment changes.^81^, ^110^ Future models could simulate different immune subtypes via gene editing/immunomodulation, advancing personalized therapy.

Model optimization also involves improved evaluation/detection techniques. Standardized clinical scoring (OSDI, TBUT, fluorescein staining) combined with statistical methods (PCA) enables comprehensive disease assessment/typing.^148^ Tear biomarker/proteomic analysis across species aids model selection; pig models are highly translatable due to tear protein similarity to humans.^92^ Advancing detection technologies will further refine model diagnosis/efficacy evaluation.

In summary, DED animal model optimization/diversification is transitioning from single‐etiology to complex multifactorial models, encompassing NHP development, immune/neural mechanism integration, and multi‐indicator evaluation systems. These advances will significantly deepen DED pathological understanding and novel therapy development, laying a solid foundation for clinical translation.

#### 3.6.3 3R principles and alternative model exploration

Growing ethical and animal welfare concerns underscore the importance of the 3R principles (Replacement, Reduction, Refinement) in DED animal model research. Alternative model exploration focuses on in vitro/ex vivo systems and computer simulation/AI‐assisted design to minimize live animal use.

In vitro/ex vivo models, like the porcine corneal ex vivo model, offer ethical, efficient platforms for DED pathology/drug screening. Porcine cornea structure/function resembles humans; its ex vivo model reasonably simulates corneal physiology/DED‐related inflammation, reducing live animal use per the Replacement principle.^156^ However, ex vivo models lack systemic immune and neural inputs, and their viability is limited to days. Computational models, though excellent for hypothesis generation and drug screening prioritization, depend heavily on the quality of input data and cannot fully replicate complex biological interactions.^157^ Computer simulation and AI technologies offer new avenues. Using big data, ML, and bioinformatic simulations, researchers can predict drug mechanisms/toxicity and optimize experiments before in vitro/animal testing, reducing animal numbers.^158^ These technologies improve research efficiency or accuracy, reveal complex disease mechanisms, and promote personalized therapy. Practically, combining porcine ex vivo models with AI‐assisted design enables multilevel (molecular‐to‐tissue) simulation, balancing biological realism and ethics.^159^ For example, using ex vivo corneas to assess DED drug corneal repair capacity, coupled with computer‐aided formulation/regimen optimization, represents a future DED research trend.^160^ Therefore, these alternatives currently serve as complementary tools rather than complete replacements, ideally used in a tiered strategy to reduce and refine live animal use before final in vivo validation.

These models provide essential tools for initial mechanistic studies and high‐throughput compound screening. For example, an in vitro wound healing model using human corneal epithelial (HCE‐T) cells was developed to evaluate the efficacy of allogeneic serum eye drops for DED. This model could detect the proliferative effect of serum at concentrations as low as 1% with low inter‐assay variability. Furthermore, an inflammatory DED model was constructed by stimulating cells with IL‐1β and TNF‐α to assess the inhibitory effects of serum on the expression of inflammatory factors like MMP9.^161^ This provides a means for preclinical evaluation without animal use. Additionally, nano‐delivery systems based on elastin‐like polymers loaded with antioxidant polyphenols have demonstrated excellent biocompatibility and significant intracellular ROS scavenging capacity in human corneal epithelial cells, offering a new in vitro platform for studying antioxidant therapies for DED.^162^ These 2D and 3D culture systems can simulate key ocular surface features such as barrier function, inflammatory responses, and drug interactions, effectively replacing exploratory animal experiments for certain research questions.

These systems utilize microfluidic technology to create dynamic and controllable in vitro environments that better mimic physiology. Although reports on fully integrated “ocular surface‐on‐a‐chip” or “lacrimal gland‐on‐a‐chip” platforms are still emerging in the literature, the evolution of existing models shows a clear trend toward greater complexity. For instance, the aforementioned inflammatory DED model has been adapted from static culture to a dynamic environment simulating tear film hydrodynamics to better evaluate drug effects.^161^ This shift from static to dynamic culture is a core feature of microphysiological systems, enabling the integration of physical factors like fluid shear stress to model tear flow and evaporation. Research on MSCs, which show promise for treating ocular surface diseases including DED due to their anti‐inflammatory and regenerative properties, provides a cellular foundation for future chip development.^163^

These technologies leverage existing data for prediction and analysis, significantly reducing exploratory animal testing aimed at target and lead compound discovery. In DED research, ML algorithms can integrate clinical and multi‐omics data (e.g., transcriptomics, proteomics) to build disease prediction models and identify key biomarkers. For example, design of experiment (DoE) modeling, a statistical computational method, was employed to optimize the induction conditions for MMP9 in the development of the in vitro inflammatory model, efficiently determining optimal parameters and reducing trial‐and‐error costs.^161^ In drug development, physiologically based pharmacokinetic/pharmacodynamic (PBPK/PD) modeling can simulate drug distribution, retention, and target interaction on the ocular surface, predicting the efficacy of new formulations in silico during the preclinical phase. For instance, the in vitro release and ex vivo corneal permeation studies of a cyclosporine A nanocarrier film can inform predictions of its PK behavior.^164^

In conclusion, although porcine corneal ex vivo models and other alternatives remain valuable, a tiered research strategy that prioritizes these advanced in vitro, organ‐on‐a‐chip, and in silico platforms for early‐stage hypothesis testing and screening, followed by targeted in vivo validation, represents the most effective and ethical path forward. Continuous refinement of these alternative models will be crucial for advancing DED mechanistic research and therapy development.

#### 3.6.4 Interdisciplinary integrated research

As a complex multifactorial disease, DED pathogenesis involves cross interactions across immunology, neuroscience, bioengineering, and other disciplines. Interdisciplinary research combining these fields provides new perspectives/tools for deciphering DED complexity.

Immunology reveals correlations between autoimmune diseases (rheumatoid arthritis, systemic sclerosis) and DED, implicating immune‐inflammatory responses in pathogenesis.^165^, ^166^ Neuroscience advances highlight neurosensory abnormalities—neurogenic inflammation, pain conduction defects, neural dysregulation—in DED pathophysiology.^167^, ^168^

Bioengineering introduces advanced tools. Biosensors and microfluidics enable real‐time monitoring of tear composition, ocular surface environment, and neural function. Biosensor‐embedded wearables (smart contact lenses, eyelid sensors) allow continuous DED physiological monitoring, facilitating precise diagnosis/personalized therapy.^169^ Nanotechnology/tissue engineering applications (nanocarrier antioxidants, stem cell therapy) reflect deep bioengineering‐clinical integration.^170^

Interdisciplinary integration extends beyond mechanisms to synergistic diagnosis/therapy optimization. Multidisciplinary teams (immunologists, neuroscientists, bioengineers, clinicians) advance personalized DED treatment via data sharing, joint experimental/clinical trial design. Precision subtyping/medication can be achieved by comprehensive analysis patient immune status, neural function, ocular surface biomarkers.^167^ Interdisciplinary collaboration also fosters novel therapies—mechanism‐based targeted immunosuppressants, neuromodulators, biomaterials—improving efficacy/quality of life.^171^, ^172^

Interdisciplinary approaches also benefit DED‐related systemic disease management. Collagen vascular/hematologic malignancy patients often have DED/ocular surface inflammation, necessitating rheumatology‐hematology‐ophthalmology collaboration for holistic care.^165^, ^173^ Public health/community‐level pharmacy/social work involvement strengthens DED early identification, patient education, treatment adherence, promoting comprehensive management.^174^, ^175^

In summary, interdisciplinary research integrating immunology, neuroscience, and bioengineering provides multidimensional insights into DED pathogenesis while advancing precise diagnosis/personalized therapy. This collaborative model drives theoretical breakthroughs and clinical translation, promising greater future impact on DED/comorbidities management. Sustaining interdisciplinary communication/cooperation, establishing unified data platforms/standardized workflows, is key to progressive DED interdisciplinary research.

**FIGURE 7:**
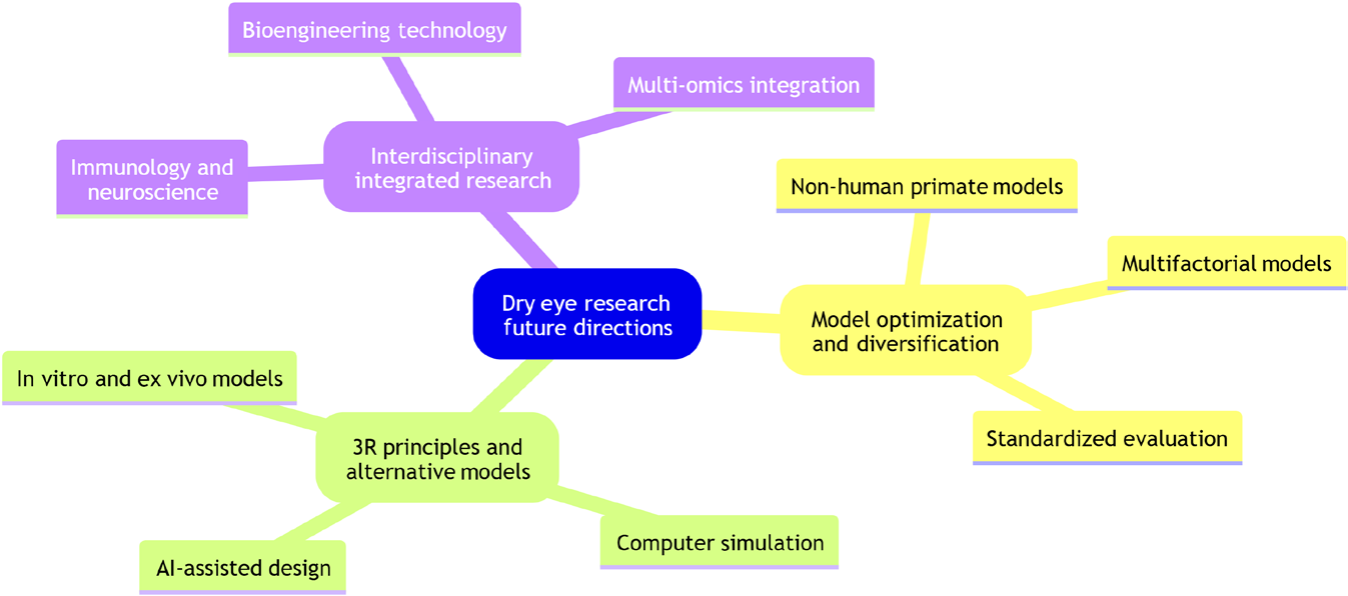
Framework for future research directions.

Figure 7 uses a mind map to outline three major future DED research trajectories: model optimization for higher fidelity, 3R principles for ethical/technological innovation, and interdisciplinary integration for field advancement via technological convergence, all oriented toward precision medicine.

## 4 DISCUSSION

The investigation of DED relies profoundly on preclinical animal models to decipher its complex pathogenesis, which spans aqueous deficiency, evaporative dysfunction, neurogenic disruption, and immune‐mediated inflammation. As systematically reviewed, the existing diverse repertoire of models—from single‐pathology to multifactorial integrated states—provides indispensable and complementary tools. They enable the simulation of varied clinical presentations and have established a solid groundwork for basic mechanistic research and therapeutic screening. From an expert standpoint, distinct models have played unique roles: aqueous‐deficient models elucidate the lacrimal gland‐inflammatory axis, evaporative models replicate ocular surface environmental stress, and neurogenic models uncover critical neural regulatory mechanisms.

However, a critical synthesis of findings across these models reveals both convergent insights and important discrepancies. Convergently, studies consistently underscore the central role of NF‐κB‐driven inflammation and highlight the intricate neuro‐immune cross‐talk in disease perpetuation. Yet, discrepant findings, such as the variable contribution of specific T‐helper cell subsets (e.g., Th17) between autoimmune‐driven Sjögren's syndrome models and environmentally induced desiccating stress models, are equally informative. These differences often stem from model‐specific etiological triggers and underscore a fundamental principle: the inherent physiological, immunological, and anatomical disparities between animals and humans preclude any single model from fully recapitulating the complexity and heterogeneity of human DED. This inherent limitation necessitates not only cautious interpretation of experimental data but also the prudent, context‐dependent selection of models aligned with specific research questions or clinical subtypes.

Despite their utility, critical translational gaps persist in current modeling approaches. The underrepresentation of clinical heterogeneity, the common omission of comorbid conditions (e.g., aging, diabetes, rheumatologic diseases), and the typical use of acute or sub‐acute induction protocols limit the ability to model chronic, progressive human DED. Consequently, the rational selection and application of animal models are paramount for enhancing translational relevance. Future research must therefore focus on refining models to bridge these gaps. This includes developing next‐generation models that incorporate aging factors, integrate comorbidities, and employ chronic induction paradigms. Furthermore, model standardization through unified protocols and evaluation criteria is crucial to improve interstudy comparability and reproducibility across laboratories.

Looking forward, the trajectory of DED preclinical research lies in strategic integration and technological innovation. The rational application of animal models must be increasingly guided by the 3Rs (Replacement, Reduction, Refinement) principles. This involves a tiered strategy: leveraging advanced in vitro platforms (e.g., 3D ocular surface models, microfluidic “eye‐on‐a‐chip” systems) for initial high‐throughput screening and mechanistic probing, followed by targeted, hypothesis‐driven in vivo validation in the most appropriate animal model. Concurrently, technological progress promises to enrich the toolkit. The development of genetically engineered large animals, the adoption of organoids and sophisticated in silico simulations, and the integration of AI for data analysis and phenotype prediction all offer avenues to create more precise and human‐relevant pathological simulation platforms.

Collectively, although animal models will continue their central role in the DED research ecosystem for deconvoluting disease complexity and assessing therapies, their future value is maximized within an integrated framework. By judiciously balancing model strengths and weaknesses, championing interdisciplinary convergence, and strategically complementing in vivo work with human‐relevant in vitro and in silico tools, the field can accelerate the transition from bench to bedside. The ultimate application of this evolving preclinical paradigm is to fuel the advancement of personalized medicine in DED, enabling the identification of patient‐specific endotypes and the prediction of individualized therapeutic responses, thereby delivering more effective diagnostic and therapeutic solutions.

As highlighted in this review, critical gaps remain, including the underrepresentation of clinical heterogeneity, comorbidities, and aging factors in current models. Future efforts must therefore focus on developing next‐generation models that integrate these complexities, leveraging advancements in gene editing, multi‐omics, and bioengineering.

## 5 CONCLUSION

In summary, this review has systematically categorized existing preclinical animal models of dry eye disease, detailing their methodologies, characterizing their pathological outputs, and evaluating their applications in mechanistic discovery and therapeutic development. The key conclusion is that although these models are indispensable, their informative value is maximized only through critical and context‐aware selection. Future progress depends on embracing a multimodal preclinical strategy that innovatively combines refined animal models with sophisticated human‐cell‐based systems and computational tools, all while adhering to ethical principles. This integrated approach holds the greatest promise for generating translatable knowledge that will ultimately improve the management and quality of life for patients with DED.

## AUTHOR CONTRIBUTIONS

**Jinshen Liu:** Conceptualization; data curation; formal analysis; funding acquisition; investigation; methodology; project administration; resources; software; supervision; validation; visualization; writing – original draft; writing – review and editing. **Jiaqi Chen:** Conceptualization; data curation; formal analysis; funding acquisition; investigation; methodology; project administration; resources; software; supervision; validation; visualization; writing – original draft. **Zhonghui Li:** Conceptualization; data curation; formal analysis; funding acquisition; investigation; methodology; project administration; resources; software; supervision; validation; visualization. **Jiamin Zhou:** Conceptualization; data curation; formal analysis; funding acquisition; investigation; methodology; project administration; resources; software; supervision; validation; visualization. **Hui Zhang:** Conceptualization; data curation; formal analysis; funding acquisition; investigation; methodology; project administration; resources; software; supervision. **Yujia Yu:** Conceptualization; data curation; formal analysis; funding acquisition; investigation; methodology; project administration; resources; software; supervision. **Feng Cheng:** Conceptualization; data curation; formal analysis; funding acquisition; investigation; methodology; project administration; resources; software. **Jie Bao:** Conceptualization; data curation; formal analysis; funding acquisition; investigation; methodology; resources; software. **Chunhan Feng:** Conceptualization; data curation; formal analysis; funding acquisition; investigation; methodology; project administration; resources; software. **Xiangqu Yu:** Conceptualization; data curation; formal analysis; funding acquisition; investigation; methodology; project administration; resources; software. **Zhao Xia:** Conceptualization; data curation; formal analysis; funding acquisition; investigation; methodology; project administration; resources; software. **Rao Ding:** Conceptualization; data curation; formal analysis; funding acquisition; investigation; methodology; project administration; resources; software. **Xiang Li:** Conceptualization; data curation; formal analysis; funding acquisition; investigation; methodology; project administration; resources; software; supervision; validation; visualization.

## FUNDING INFORMATION

All the authors appreciate the support of the (1) Sichuan Provincial Department of Science and Technology Natural Science Foundation General Program: 2024NSFS0726, (2) Joint Innovation Fund of Commission Institutions (Key Project): WXLH202402009, (3) Joint Innovation Fund of Commission Institutions (Free Exploration Project) WXLH202403095, and (4) Sichuan Provincial Central Administration General Project: 2024MS549.

## CONFLICT OF INTEREST STATEMENT

The authors declare no competing interests.

## ETHICS STATEMENT

As this is a review article, ethics approval and consent to participateare not applicable.

## Data Availability

No datasets were generated or analyzed during the current study.
